# Low-Dose Deoxynivalenol Induces Subclinical Multi-Organ Toxicity in Weaned Piglets

**DOI:** 10.3390/toxins18020075

**Published:** 2026-02-02

**Authors:** Ying Liu, Sunlin Luo, Xinchun Zou, Wenjun He, Ruiqi Tan, Yongpeng Jin, Gaoyi Liu, Qiaomin Duan, Wenjun Yang, Yiqiang Chen

**Affiliations:** 1 State Key Laboratory of Animal Nutrition and Feeding, College of Animal Science and Technology, China Agricultural University, Beijing 100193, China; liuying01@cau.edu.cn (Y.L.); luosunlin@cau.edu.cn (S.L.); hewenjun0525@163.com (W.H.); tanruiqi@cau.edu.cn (R.T.); jinyp@cau.edu.cn (Y.J.); liugaoyi@cau.edu.cn (G.L.); xiaoduo@cau.edu.cn (Q.D.); ywj16@sina.com (W.Y.); 2 College of Life Sciences, Wuhan University, Wuhan 430072, China; 2023302041086@whu.edu.cn

**Keywords:** deoxynivalenol, weaned piglets, growth performance, intestinal health, reproductive development

## Abstract

Deoxynivalenol (DON) is a common mycotoxin in cereal crops such as corn, wheat, and their processed products. It can cause feed refusal and growth retardation in piglets. This study systematically evaluated the effects of dietary exposure to purified DON at low doses of 0.25, 0.5, 1.0, and 2.0 mg/kg on growth performance, blood biochemistry, antioxidant capacity, immune function, intestinal health, and reproductive development in female weaned piglets over a 42-day period. Although dietary exposure to 0.25–2.0 mg/kg of DON did not significantly affect growth performance, it induced subclinical multi-organ toxicity. Notably, decreased platelet count (PLT) at 0.25–2.0 mg/kg and increased serum alanine aminotransferase (ALT) activity at 2.0 mg/kg were observed. DON exposure also impaired antioxidant function with reduced serum total antioxidant capacity (T-AOC) at 0.25–2.0 mg/kg, and elevated malondialdehyde (MDA) content in the jejunum and ileum at 0.5–2.0 mg/kg. Furthermore, at all doses tested (0.25–2.0 mg/kg), DON suppressed anti-inflammatory cytokine interleukin-10 (IL-10) levels in both serum and intestine, reduced duodenal villus height (VH), and decreased serum follicle-stimulating hormone (FSH) and luteinizing hormone (LH) levels. Additionally, histopathological injuries of liver, kidney, duodenum, jejunum, ileum, uterus and ovaries were also observed at doses of 1.0–2.0 mg/kg. In summary, this study confirms the multi-organ toxicity of low-dose DON in piglets. Our findings suggest that DON concentrations in pig feed should be more strictly controlled and highlight the importance of considering subclinical health endpoints, such as oxidative stress markers and immune parameters, in future risk assessments of mycotoxin exposure.

## 1. Introduction

Deoxynivalenol (DON), a secondary metabolite produced by *Fusarium* fungi such as *Fusarium graminearum*, *Fusarium culmorum*, *Fusarium nivale*, and *Fusarium oxysporum*, belongs to the trichothecene group of mycotoxins [1,2]. It tends to proliferate in warm and humid environments and is one of the most widespread mycotoxin contaminants globally, frequently found in cereal crops such as corn, barley, and wheat, as well as their processed derivatives [3]. Corn is one of the major food crops and also a main raw material for animal feed. Therefore, DON is often detected in animal feed. A global mycotoxin survey analyzing 74,821 feed and feed raw material samples from 100 countries (2008–2017) revealed that 88% of samples contained at least one mycotoxin, with DON showing the highest detection rate of 64% and a median contamination level of 388 μg/kg [4]. Similarly, a Brazilian study examining 1749 feed and feed raw material samples (2017–2021) found that 97% were contaminated with at least one mycotoxin, with DON detected in 67.8% of samples at a median level of 446 μg/kg [5]. These findings highlight the significant contamination risk posed by DON in animal feed. Notably, DON exhibits remarkable chemical stability, with strong heat and acid resistance, allowing it to maintain structural integrity even under high-temperature processing and acidic conditions. Consequently, conventional feed processing methods fail to degrade DON effectively, which enables its entry into the food chain and poses health risks to both animals and humans [6,7].

Relevant studies have shown that DON exhibits both acute and chronic toxicity, damaging multiple target organs in animals and demonstrating various toxic effects, such as intestinal toxicity [8], neurotoxicity [9], hepatotoxicity [10], reproductive toxicity [11,12], immunotoxicity [13], cytotoxicity [14], and oxidative stress [15]. The primary manifestations of acute and chronic poisoning in piglets include reduced feed intake, anorexia, diarrhea, growth retardation, increased susceptibility to diseases, and even acute death [2]. Research has shown that weaned piglets fed diets containing 1.0 and 3.0 mg/kg DON developed duodenal edema, damage to jejunal villus epithelial cells, and lymphocyte infiltration in the ileum, accompanied by aggravated lipid peroxidation and increased serum lipopolysaccharide levels, indicating that DON can disrupt intestinal barrier function and induce oxidative stress [16]. Another study confirmed that 4 mg/kg DON altered the morphology of the jejunum and ileum in piglets, while also exacerbating intestinal inflammation by upregulating the gene and protein expression levels of pro-inflammatory cytokines in intestinal tissues, thereby reducing growth performance [17]. Besides, DON has also been shown to induce oxidative stress and inflammatory responses by activating the nuclear factor kappa-B signaling pathway, leading to hepatocyte apoptosis and hepatic inflammatory lesions [18].

After ingestion, DON can rapidly cross the intestinal barrier and be absorbed by animals, entering the bloodstream and distributing to various tissues and organs, causing organ damage [19]. Compared to poultry and ruminants, pigs are more sensitive to DON [2]. Studies have shown that DON can be detected in the blood of pigs within 30 min after oral administration, and the duodenum can absorb DON quickly and efficiently [20,21]. The absorption of DON varies among different animal species, with research reporting the following absorption order: pigs (82%) > chickens (19%) > sheep (5.9–9.9%) > cattle (1%) [22,23,24,25]. These differences in absorption rates may be related to the distribution of intestinal microorganisms in different animals. Substantial differences in microbial community structure and density along the gastrointestinal tract have been verified [26], which could, in turn, influence the local luminal environment and the processing of dietary contaminants, potentially affecting their absorption efficiency. Numerous exposure studies have demonstrated that DON tends to accumulate in tissues. In edible tissues of pigs, the residual levels of DON and its less toxic metabolite, deepoxy-deoxynivalenol (DOM-1), follow this order: kidneys > liver > muscles > spleen > fat [27,28].

Given the severe adverse effects of DON on animal and human health, many agriculturally advanced countries have set maximum permitted levels for DON in animal feed to mitigate the risk of mycotoxicosis. European Union regulations stipulate that the maximum permitted level of DON in pig compound feed must not exceed 0.9 mg/kg [29]. In America and China, the upper limit of DON in pig feed is set at 1 mg/kg [30,31]. Notably, most domestic and international studies have focused on the effects of high doses of naturally contaminated DON on weaned piglets. However, these results are susceptible to interference from other coexisting contaminants in the feed and the reduced nutritional quality resulting from fungal proliferation. Consequently, it remains challenging to accurately evaluate the specific toxicological effects of DON molecules on piglets. Additionally, low-dose DON contamination in feed is more prevalent globally, and the high sensitivity of pigs to DON suggests that even low-dose DON exposure may cause harm to piglets. Currently, research on the health effects of low-dose DON in piglets remains limited, lacking comprehensive and systematic assessment. Therefore, this study aims to systematically investigate the effects of low doses of purified DON (0.25, 0.5, 1.0, and 2.0 mg/kg) on growth performance, blood biochemistry, antioxidant capacity, immune function, intestinal health, and reproductive development in female weaned piglets. Our experimental doses (0.25, 0.5, 1.0, and 2.0 mg/kg) were selected to cover both common contamination ranges (0.25–0.5 mg/kg) and higher exposure scenarios (1.0–2.0 mg/kg) that align with or slightly exceed current regulatory limits. This design underscores the real-world relevance of our study and highlights the need to assess subclinical effects at realistically low contamination levels. The findings of this study could provide a scientific basis for optimizing mycotoxin control strategies in feed and informing potential revisions to regulatory limits.

## 2. Results

### 2.1. Effects of DON on Growth Performance in Piglets

As shown in Table 1, dietary DON contamination at all tested levels (0.25, 0.5, 1.0, and 2.0 mg/kg) did not exert any significant impact (*p* > 0.05) on the growth performance of piglets throughout the 42-day experimental period. This indicates that low-dose purified DON exposure did not cause overt growth retardation in piglets under the conditions of this study.

### 2.2. Effects of DON on Hematological and Biochemical Parameters in Piglets

Dietary DON exposure significantly altered specific hematological and biochemical parameters in piglets. The complete data are presented in Supplementary Tables S1 and S2, while statistically significant findings are summarized in Table 2. Hematologically, a marked decrease in platelet count (PLT) was observed across all DON-treated groups on day 28 (*p* < 0.01). By day 42, the platelet distribution width (PDW) and mean platelet volume (MPV) were significantly reduced in the DON0.25, DON0.5, and DON1.0 groups (*p* < 0.01). These alterations in platelet indices indicate a potential disturbance in thrombopoiesis or platelet activation status. Biochemically, the DON0.5 group exhibited a significant increase in aspartate aminotransferase (AST) activity on both day 28 (*p* < 0.01) and day 42 (*p* < 0.05). Additionally, alanine aminotransferase (ALT) activity was higher in the DON2.0 group than in the CON group on day 42 (*p* < 0.05). The increased AST and ALT activities suggest subclinical liver damage induced by DON exposure.

### 2.3. Effects of DON on Serum Lipid Profiles in Piglets

The effects of dietary DON exposure on serum lipid profiles are summarized in Table 3. On day 14, serum triglyceride (TG) concentration was significantly higher in the DON0.25 group than in the CON group (*p* < 0.05). On day 28, serum high-density lipoprotein cholesterol (HDL-C) concentration was lower in the DON0.25 and DON0.5 groups (*p* < 0.05), whereas the DON2.0 group had a higher serum low-density lipoprotein cholesterol (LDL-C) concentration (*p* < 0.05). By day 42, serum HDL-C concentration remained lower in the DON0.5 group (*p* < 0.05). Collectively, these lipid profile changes indicate disrupted lipid homeostasis and a pro-atherogenic tendency in DON-exposed piglets.

### 2.4. Effects of DON on Antioxidant Function in Piglets

Serum antioxidant status results are shown in Table 4. DON significantly reduced serum total antioxidant capacity (T-AOC) on days 14 and 42 (*p* < 0.01). Specifically, the DON0.25, DON1.0, and DON2.0 groups exhibited lower T-AOC on day 28 (*p* < 0.01). Conversely, serum malondialdehyde (MDA) concentration was increased in the DON1.0 group on day 14 (*p* < 0.05).

**Figure 1:**
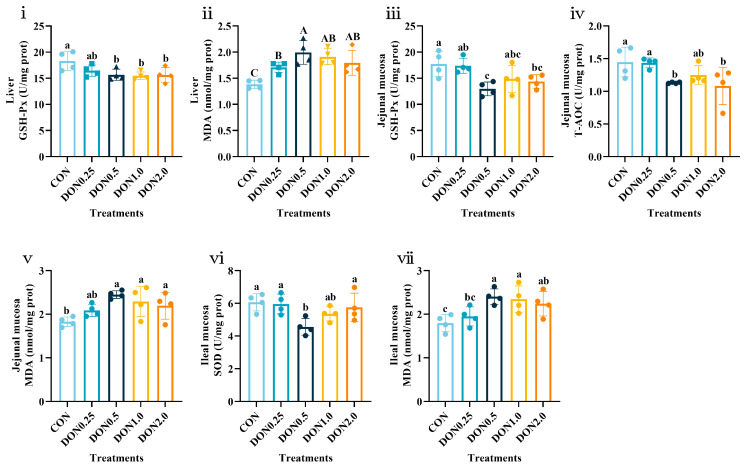
Effects of dietary DON exposure on antioxidant function in the liver and intestine of piglets. (**i**) Glutathione peroxidase (GSH-Px) activity and (**ii**) malondialdehyde (MDA) content in the liver. (**iii**) GSH-Px activity, (**iv**) total antioxidant capacity (T-AOC), and (**v**) MDA content in the jejunal mucosa. (**vi**) superoxide dismutase (SOD) activity and (**vii**) MDA content in the ileal mucosa. Data are presented as means ± SD, *n* = 4. A–C, different letters mean a statistical difference (*p* < 0.01). a–c, different letters mean a statistical difference (*p* < 0.05).

DON exposure also affected antioxidant function in the liver and intestine (Supplementary Table S3; Figure 1). In the liver, DON exposure (0.5–2.0 mg/kg) suppressed glutathione peroxidase (GSH-Px) activity (*p* < 0.05) and increased MDA content (*p* < 0.01). In the jejunal mucosa, GSH-Px activity and T-AOC content were reduced in the DON0.5 and DON2.0 groups (*p* < 0.05), while MDA content was increased by 0.5–2.0 mg/kg DON exposure (*p* < 0.05). In the ileal mucosa, superoxide dismutase (SOD) activity was lower in the DON0.5 group (*p* < 0.05), and MDA content was higher in the DON0.5, DON1.0 and DON2.0 groups (*p* < 0.05). Together, the systemic reduction in T-AOC, the tissue-specific suppression of the key antioxidant enzymes GSH-Px and SOD activity, and the increased MDA content demonstrate that low-dose DON exposure induces a state of systemic and multi-organ oxidative stress in piglets.

### 2.5. Effects of DON on Immune Performance in Piglets

Serum immunoglobulin levels are shown in Supplementary Table S4. Dietary DON exposure did not affect serum immunoglobulin A (IgA), immunoglobulin G (IgG), or immunoglobulin M (IgM) levels at any stage (*p* > 0.05). However, liver IgM content was reduced in the DON1.0 and DON2.0 groups (*p* < 0.05, Supplementary Figure S1).

**Figure 2:**
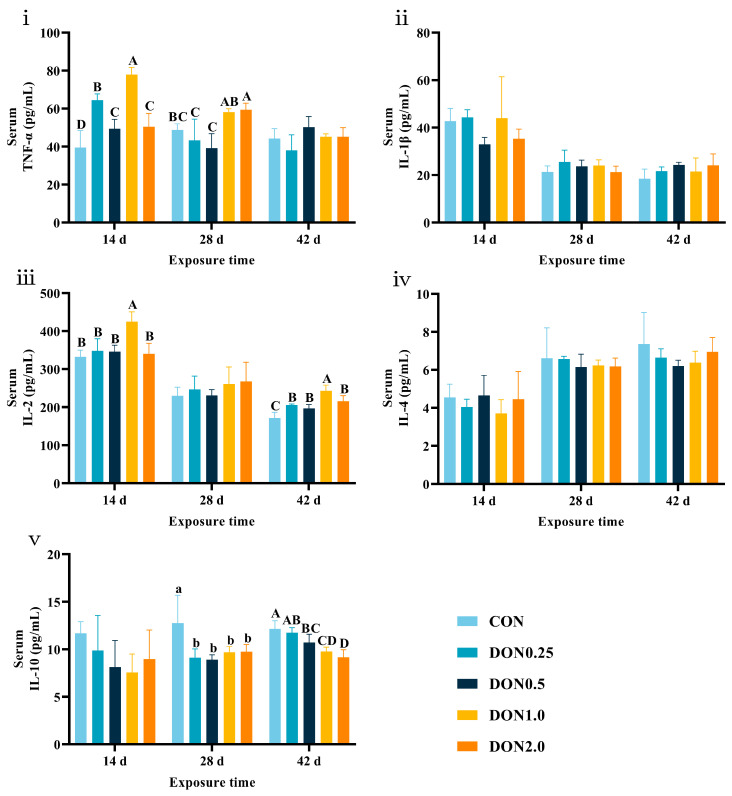
Effects of dietary DON exposure on serum immune cytokine levels of piglets. (**i**) Tumor necrosis factor-α (TNF-α), (**ii**) Interleukin-1β (IL-1β), (**iii**) Interleukin-2 (IL-2), (**iv**) Interleukin-4 (IL-4), and (**v**) Interleukin-10 (IL-10). Data are presented as means ± SD, *n* = 4. A–D, different letters mean a statistical difference (*p* < 0.01). a, b, different letters mean a statistical difference (*p* < 0.05).

Serum cytokine dynamics are shown in Figure 2. On day 14, serum tumor necrosis factor-α (TNF-α) levels were increased in all DON-treated groups (*p* < 0.01), and interleukin-2 (IL-2) levels were increased in the DON1.0 group (*p* < 0.01). On day 28, serum TNF-α levels were higher in the DON2.0 group (*p* < 0.01), while interleukin-10 (IL-10) levels were lower in all DON-treated groups (*p* < 0.05). On day 42, serum IL-2 levels were increased in all DON-treated groups (*p* < 0.01), and IL-10 levels were decreased in the DON 0.5, DON 1.0, and DON 2.0 groups (*p* < 0.01).

In the liver (Supplementary Figure S2), TNF-α level was higher in the DON0.5 group than in the CON group (*p* < 0.01). No significant effects were observed on liver IL-1β, IL-2, IL-4 or IL-10 levels (*p* > 0.05).

**Figure 3:**
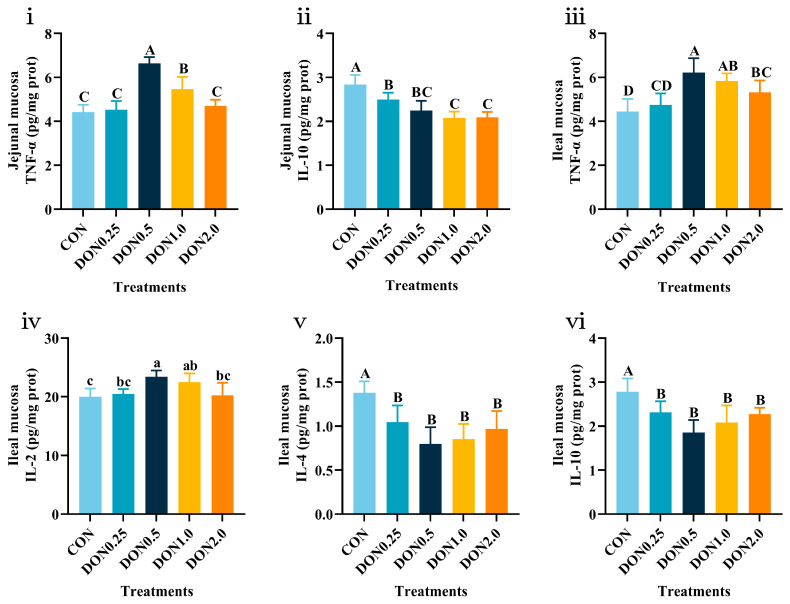
Effects of dietary DON exposure on intestinal mucosal immune cytokine levels of piglets. (**i**) Tumor necrosis factor-α (TNF-α) and (**ii**) Interleukin-10 (IL-10) levels in the jejunal mucosa. (**iii**) TNF-α, (**iv**) Interleukin-2 (IL-2), (**v**) Interleukin-4 (IL-4) and (**vi**) IL-10 levels in the ileal mucosa. Data are presented as means ± SD, *n* = 4. A–D, different letters mean a statistical difference (*p* < 0.01). a–c, different letters mean a statistical difference (*p* < 0.05).

The effects on intestinal mucosal cytokine levels are provided in Supplementary Table S5, with significant results detailed in Figure 3. Dietary DON exposure did not affect the levels of immune cytokines in the duodenal mucosa (*p* > 0.05). In the jejunal mucosa, TNF-α levels were increased in the DON0.5 and DON1.0 groups (*p* < 0.01), and IL-10 levels were decreased in all DON-treated groups (*p* < 0.01). In the ileal mucosa, TNF-α levels were elevated in the DON0.5, DON1.0, and DON2.0 groups (*p* < 0.01), and IL-2 levels were higher in the DON0.5 and DON1.0 groups (*p* < 0.05). Both IL-4 and IL-10 levels were reduced in all DON-treated groups (*p* < 0.01), while no significant effect was found on IL-1β levels (*p* > 0.05). Thus, these findings indicate that low-dose DON exposure did not affect systemic humoral immunity but induced a distinct pro-inflammatory state, characterized by elevated pro-inflammatory cytokines and suppressed anti-inflammatory cytokines, particularly in the intestinal mucosa.

### 2.6. Effects of DON on Intestinal Barrier Function in Piglets

**Figure 4:**
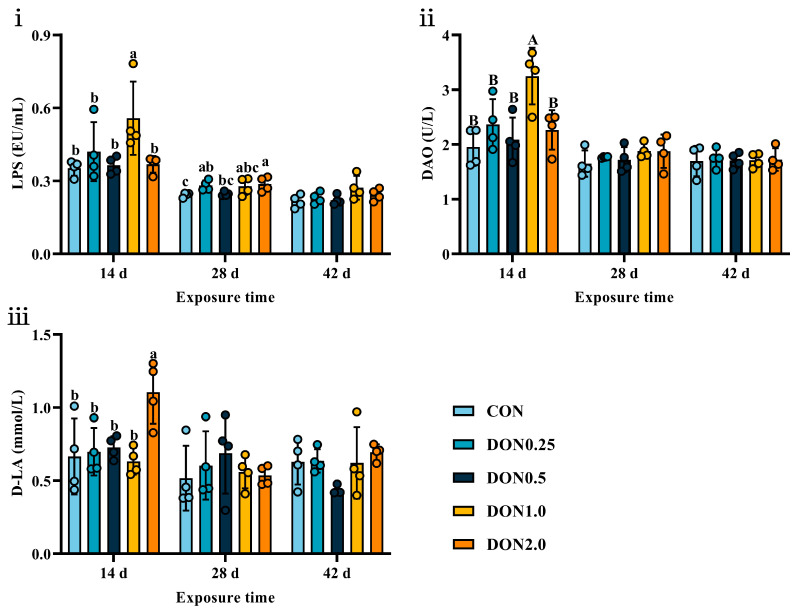
Effects of dietary DON exposure on intestinal barrier function of piglets. (**i**) Lipopolysaccharide (LPS), (**ii**) diamine oxidase (DAO), and (**iii**) D-lactic acid (D-LA) levels in the serum. Data are presented as means ± SD, *n* = 4. A, B, different letters mean a statistical difference (*p* < 0.01). a–c, different letters mean a statistical difference (*p* < 0.05).

As shown in Figure 4, dietary DON exposure induced limited alterations in serum markers of intestinal barrier integrity. On day 14, serum levels of lipopolysaccharide (LPS, *p* < 0.05) and diamine oxidase (DAO, *p* < 0.01) were elevated in the DON1.0 group, and D-lactic acid (D-LA) level was increased in the DON2.0 group (*p* < 0.05). By day 28, serum LPS levels were higher in the DON0.25 and DON2.0 groups (*p* < 0.05). Notably, these effects were transient, as no significant differences in these markers were observed by day 42 (*p* > 0.05). This result indicates that DON exposure caused a temporary, subclinical compromise of intestinal barrier integrity in piglets.

### 2.7. Intestinal Morphology and Histopathology Analysis

**Figure 5:**
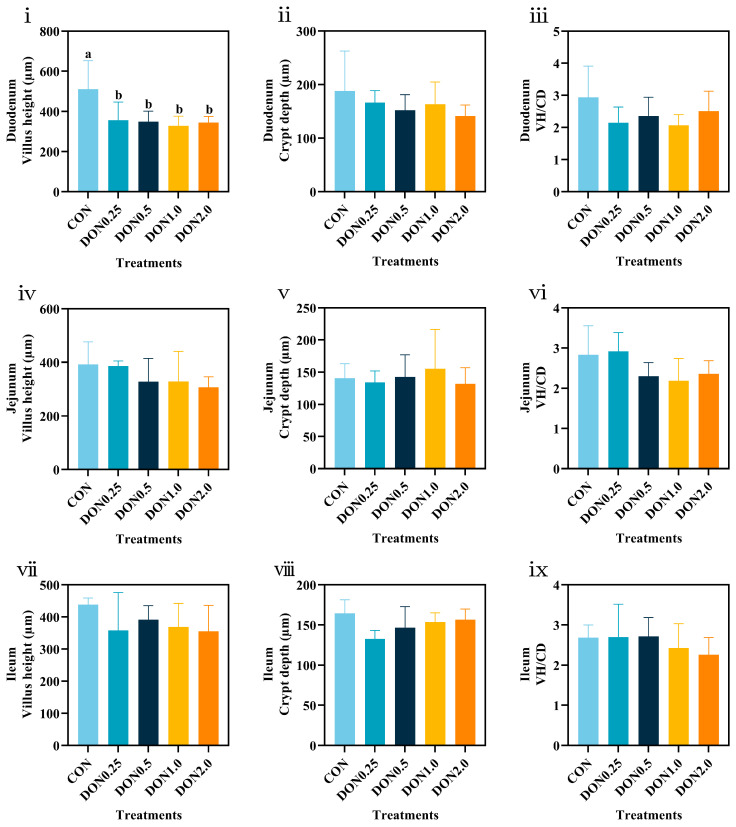
Effects of dietary DON exposure on intestinal morphology in piglets. (**i**–**iii**) Villus height (VH), crypt depth (CD), and the villus height-to-crypt depth ratio (VH/CD) in the Duodenum, respectively. (**iv**–**vi**) VH, CD, and VH/CD in the jejunum, respectively. (**vii**–**ix**) VH, CD, and VH/CD in the ileum, respectively. Data are presented as means ± SD, *n* = 4. a, b, different letters mean a statistical difference (*p* < 0.05).

Quantitative analysis of intestinal morphology is shown in Figure 5. All DON-treated groups exhibited a reduction in duodenal villus height (VH) compared to the CON group (*p* < 0.05). In contrast, dietary DON exposure had no significant effects on the VH, crypt depth (CD), or the villus height-to-crypt depth ratio (VH/CD) in the jejunum or ileum (*p* > 0.05). These results demonstrate that low-dose DON exposure specifically impairs duodenal morphology in piglets.

**Figure 6:**
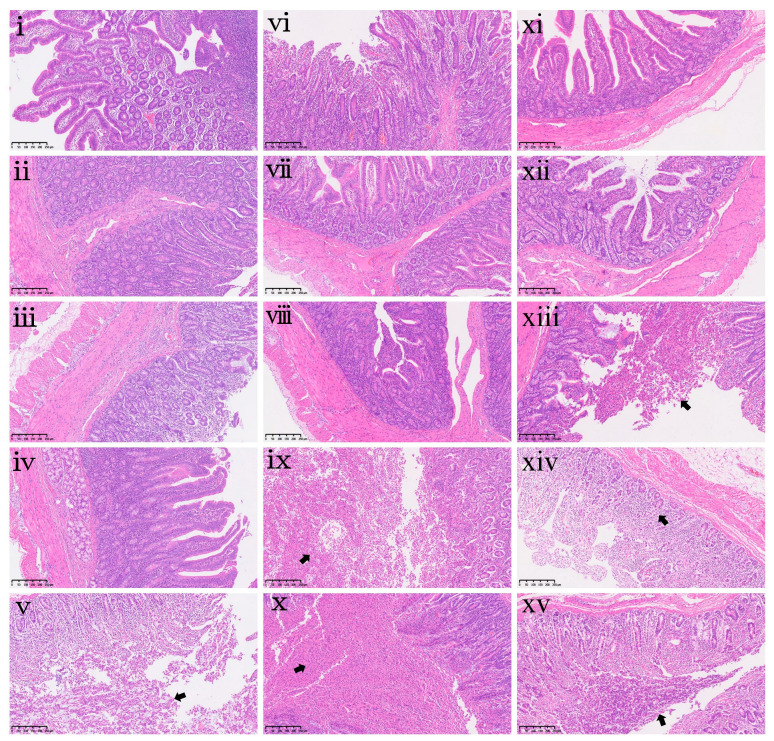
Intestinal histopathological changes in piglets following dietary DON exposure. (**i**–**v**) Duodenum sections, (**vi**–**x**) jejunum sections, and (**xi**–**xv**) ileum sections from piglets in the CON, DON0.25, DON0.5, DON1.0 and DON2.0 groups, respectively. Black arrows in (**v**) indicate moderate sloughing of mucosal components, including epithelium, stromal cells, and goblet cells; in (**ix**,**x**), moderate to severe epithelial detachment and accumulation; in (**xiii**,**xv**), mild epithelial detachment and accumulation; and in (**xiv**), moderate mononuclear cell hyperplasia. All specimens were examined at 100× magnification. Scale bar = 250 μm.

To further characterize the intestinal injury, histopathological examination was performed (Figure 6). This qualitative assessment revealed substantial damage that was not fully captured by the morphometric measurements. In the duodenum, the DON2.0 group showed moderate sloughing of mucosal components (including epithelium, stromal cells, and goblet cells) into the lumen (Figure 6v). In the jejunum, moderate to severe epithelial detachment and accumulation were observed in the DON1.0 and DON2.0 groups (Figure 6ix,x). In the ileum, histopathological examination revealed mild epithelial detachment and accumulation in the DON0.5 and DON2.0 groups (Figure 6xiii,xv). Additionally, the DON1.0 group exhibited moderate mononuclear cell hyperplasia in the lamina propria (Figure 6xiv).

### 2.8. Effects of DON on Organ Indices and Hepatorenal Histopathology in Piglets

**Figure 7:**
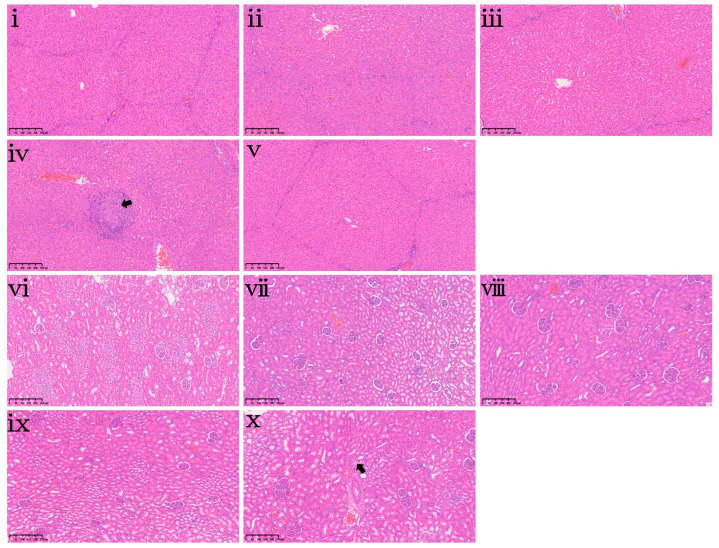
Hepatorenal histopathological changes in piglets following 42-day dietary DON exposure. (**i**–**v**) Liver sections and (**vi**–**x**) kidney sections from piglets in the CON, DON0.25, DON0.5, DON1.0 and DON2.0 groups, respectively. Black arrows indicate mild focal necrosis of hepatocytes in (**iv**) and mild vacuolation of renal tubules in (**x**). All specimens were examined at 100× magnification. Scale bar = 250 μm.

At the end of the 42-day feeding trial, the effects of dietary DON exposure on organ indices and hepatorenal histopathology were evaluated and are presented in Table 5 and Figure 7, respectively. Dietary DON exposure did not affect the indices of the heart, liver, spleen, lungs, or kidneys (Table 5, *p* > 0.05). This indicates that DON exposure in this study did not lead to measurable changes in organ mass. However, histopathological examination revealed mild focal necrosis of hepatocytes in the liver of the DON1.0 group (Figure 7iv) and mild vacuolation of renal tubules in the kidney of the DON2.0 group (Figure 7x).

### 2.9. Effects of DON on Reproductive Development in Piglets

**Figure 8:**
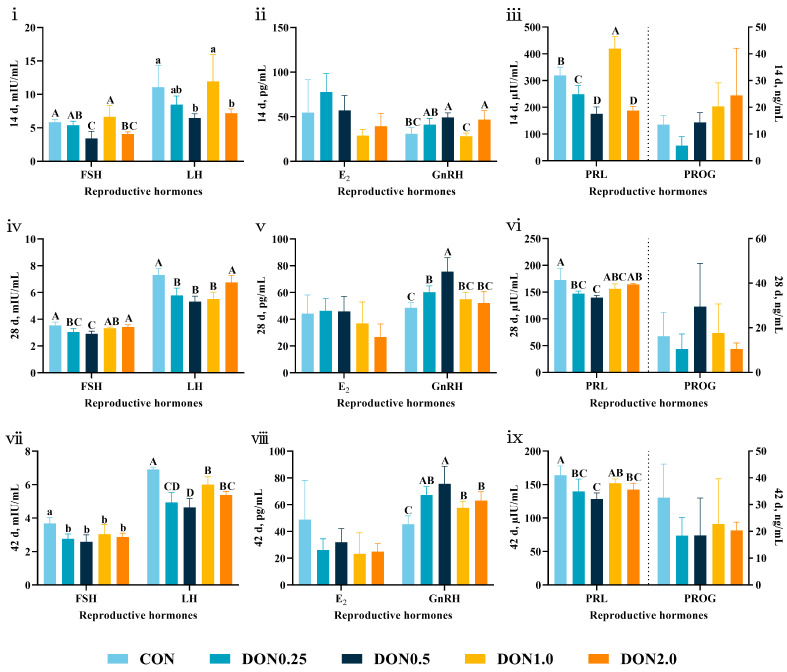
Effects of dietary DON exposure on serum reproductive hormone levels in piglets. Serum levels of follicle-stimulating hormone (FSH), luteinizing hormone (LH), estradiol (E_2_), gonadotropin-releasing hormone (GnRH), prolactin (PRL), and progesterone (PROG) were determined on (**i**–**iii**) day 14, (**iv**–**vi**) day 28, and (**vii**–**ix**) day 42. The dashed lines demarcate the two y-axes, which represent serum levels of PRL on the left and PROG on the right. Data are presented as means ± SD, *n* = 4. A–D, different letters mean a statistical difference (*p* < 0.01). a, b, different letters mean a statistical difference (*p* < 0.05).

Serum reproductive hormone levels were altered following DON exposure (Figure 8). On day 14, serum follicle-stimulating hormone (FSH) and luteinizing hormone (LH) levels were decreased in the DON0.5 and DON2.0 groups (*p* < 0.01 and *p* < 0.05, respectively), while gonadotropin-releasing hormone (GnRH) level was increased (*p* < 0.01). Serum prolactin (PRL) level was also reduced in the DON0.25, DON0.5, and DON2.0 groups (*p* < 0.01). A similar trend was observed on day 28. Serum FSH and PRL levels were lower in the DON0.25 and DON0.5 groups (*p* < 0.01), and GnRH level was higher (*p* < 0.01). Additionally, serum LH level was suppressed in the DON0.25, DON0.5, and DON1.0 groups (*p* < 0.01). By day 42, all DON-treated groups had reduced serum FSH (*p* < 0.05) and LH (*p* < 0.01) levels, and GnRH level was increased (*p* < 0.01). Serum PRL level was also lower in the DON0.25, DON0.5, and DON2.0 groups (*p* < 0.01). These consistent alterations across multiple reproductive hormones demonstrate that low-dose DON exposure induces significant endocrine disruption in weaned piglets.

**Figure 9:**
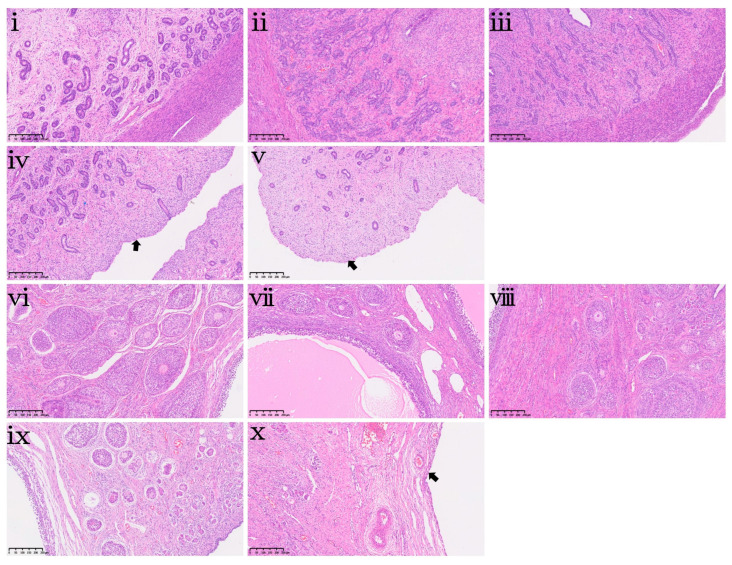
Histopathological changes in the uterus and ovary of piglets following 42-day dietary DON exposure. (**i**–**v**) Uterus sections and (**vi**–**x**) ovary sections from piglets in the CON, DON0.25, DON0.5, DON1.0 and DON2.0 groups, respectively. Black arrows indicate severe endometrial epithelial cell shedding in (**iv**,**v**), and indicate follicular cysts in (**x**). All specimens were examined at 100× magnification. Scale bar = 250 μm.

Histomorphological analysis of the uterus and ovary revealed substantial damage (Figure 9). Severe endometrial epithelial cell shedding was observed in the uterine tissue of the DON1.0 and DON2.0 groups (Figure 9iv,v). Moreover, follicular cysts were present in the ovarian tissue of the DON2.0 group (Figure 9x).

## 3. Discussion

Several studies have demonstrated that DON-contaminated diets can significantly impair the growth performance of piglets. Liao et al. [17] reported that weaned piglets fed a diet containing 4 mg/kg DON for only 14 days exhibited significant reductions in final BW, ADG, and ADFI, along with an increased F:G. Consistent with this, another study found that prolonged consumption (28 days) of a naturally contaminated diet containing 2.5 mg/kg DON also led to significant decreases in BW and ADG [32]. However, in contrast to our findings, the aforementioned studies utilized naturally contaminated feedstuffs, where the observed effects could be amplified by the synergistic interactions of other co-occurring mycotoxins. Our study, employing purified DON, clearly demonstrates that low doses (0.25–2.0 mg/kg) did not significantly affect the growth performance of piglets. This discrepancy suggests that the direct growth-inhibiting effect of low-dose pure DON might be limited, and the combined toxicity of multiple pollutants present in naturally contaminated materials could be a critical factor driving the significant growth suppression observed in previous studies. To fully elucidate the potential risks of long-term, low-level DON exposure on growth and health, further investigations with larger sample sizes and extended durations are warranted.

Beyond growth performance, this study evaluated the physiological impact of DON by examining hematological, biochemical, and lipid metabolic parameters. While hematological indices remained largely unaffected on day 14, significant alterations emerged by days 28 and 42, including reduced PLT, PDW, and MPV. This delayed effect suggests a potential for cumulative DON toxicity, leading to impaired platelet production or function over time. Concurrently, serum biochemistry indicated perturbations in organ function. Notably, significant elevations in the activities of the liver enzymes AST and ALT were observed in some groups during the mid to late stages. Serum AST and ALT activities are key biomarkers of hepatic injury, and their elevation indicates hepatocyte damage and increased membrane permeability [33]. This progression demonstrates that low-level DON exposure (0.25–2.0 mg/kg) can induce mild but measurable hepatic stress over time, corroborating a previous report [34]. Furthermore, DON exposure disrupted lipid metabolism. TG levels increased on day 14, followed by decreased HDL-C and increased LDL-C levels on days 28 and 42. This atherogenic lipid profile suggests that DON-induced liver injury or inflammation interferes with normal lipid homeostasis. This finding can be explained by the prior discovery that dietary exposure to DON at a level of 1040 μg/kg for 28 days downregulates key genes in lipid metabolism, such as Perilipin 1 (PLIN1), Perilipin 4 (PLIN4), Adiponectin (ADIPOQ), and Fatty Acid Binding Protein 4 (FABP4) in piglets [35]. Moreover, exposure to single or combined *Fusarium* mycotoxins has been shown to modify the membrane lipid profiles and serum biochemistry in the visceral organs of piglets [36]. Therefore, this integrated analysis demonstrates that subchronic exposure to DON, even at low levels, can lead to cumulative systemic effects in piglets, manifesting as impaired platelet function, mild hepatic stress, and disrupted lipid metabolism.

The observed hepatic and metabolic disturbances are likely closely linked to oxidative stress, a well-established mechanism of DON toxicity. DON disrupts the redox balance by inducing reactive oxygen species accumulation and overwhelming the antioxidant defense system [37]. This study provides direct evidence that even low-dose DON (0.25–2.0 mg/kg) is sufficient to induce systemic and local oxidative stress. We observed a sustained reduction in serum T-AOC levels, alongside decreased hepatic GSH-Px activity and increased MDA content. Critically, this oxidative insult was confirmed in the intestinal mucosa, with compromised antioxidant status (reduced GSH-Px activity and T-AOC) and elevated lipid peroxidation (increased MDA content) in the jejunum and ileum. These findings are consistent with prior reports where higher DON doses (3–4 mg/kg) suppressed serum GSH-Px and SOD activities while elevating MDA levels [34,38]. The underlying mechanism may involve DON-induced inhibition of the Akt/mTOR/4EBP1 signaling pathway, impairing protein synthesis and cellular proliferation [38], coupled with the activation of MAPK pathway and Caspase-mediated apoptotic pathways triggered by oxidative stress [39,40]. Furthermore, the resulting oxidative stress may also impair hepatic cytochrome P450 (CYP450) enzymes, which are capable of detoxifying DON [2,6]. This potential compromise in CYP450-mediated clearance could exacerbate DON retention and toxicity, though the precise mechanisms require further investigation.

In addition to metabolic and oxidative effects, DON exerts complex immunomodulatory effects, potentiating the immune response at low doses while suppressing them at high levels [41,42]. This study provides a comprehensive assessment of its impact on both humoral and cellular immunity in piglets. In contrast to a study employing a higher dose of 8 mg/kg that reported significant reductions in serum IgG and IgM concentrations [43], our findings revealed that low-level DON exposure (0.25–2.0 mg/kg) did not alter serum IgA, IgG, or IgM concentrations. However, a significant decrease in hepatic IgM concentration was observed at doses of 1.0–2.0 mg/kg, suggesting a potential for localized immunotoxicity even in the absence of a systemic humoral response. A more pronounced effect was observed on the balance between pro- and anti-inflammatory cytokines, indicating disrupted cellular immunity. We observed a systemic pro-inflammatory shift, characterized by elevated serum TNF-α levels in the early phase and increased IL-2 in the late stages, concomitant with a reduction in the anti-inflammatory cytokine IL-10. This inflammatory state was particularly evident in local tissues. While the liver and duodenum showed minimal changes, the jejunal and ileal mucosa displayed a marked inflammatory profile, with significant increases in TNF-α levels and decreases in IL-10 levels across the 0.25–2.0 mg/kg dose range. The ileum was notably sensitive, also exhibiting a reduction in IL-4 levels. The heightened susceptibility of the distal intestine implicates regional differences in immune cell distribution or gut microbiota composition. Specifically, the characteristic microbial community in the ileum may contribute to its sensitivity by influencing local barrier integrity, immune homeostasis, and xenobiotic metabolism, although the specific mechanisms require further investigation. The observed upregulation of pro-inflammatory cytokines aligns with established mechanistic studies, which demonstrate that DON activates the NF-κB pathway, thereby promoting the transcription and expression of cytokines like TNF-α and IL-1β [17,44]. Concurrent suppression of anti-inflammatory cytokines (IL-4, IL-10) further disrupts immune homeostasis [45]. Therefore, our results demonstrate that subchronic, low-level DON exposure does not induce a systemic humoral deficiency but rather triggers a distinct pro-inflammatory state, compromising immune homeostasis primarily in the systemic circulation and the distal intestinal tract, likely through mechanisms involving the dysregulation of key immunomodulatory pathways.

Given the intestine’s role as the primary barrier against dietary contaminants, it represents a key target for DON. Serum biomarkers, including LPS, DAO, and D-LA, serve as effective indicators of intestinal permeability and barrier integrity. Elevated serum levels of these markers signify impaired gut barrier function [46,47]. In this study, alterations in these markers were primarily observed on days 14 and 28. Specifically, the DON1.0 group showed elevated LPS and DAO levels, and the DON2.0 group exhibited increased D-LA level. However, these effects were transient, potentially due to enhanced intestinal adaptation over time. These results suggest that DON at 1.0–2.0 mg/kg only moderately and temporarily compromises gut barrier function. Our findings are consistent with studies reporting increased plasma DAO and D-LA levels in piglets exposed to 2.65 and 4 mg/kg DON [38,48], but indicate a weaker and non-persistent impact at lower doses. Moreover, serum LPS and DAO levels were increased at 1.0 mg/kg DON but showed a return toward control levels at 2.0 mg/kg on day 14. This pattern may reflect a compensatory or adaptive response of the intestinal epithelium under sustained DON exposure. Beyond barrier function, intestinal health relies on structural integrity, which is assessed through VH, CD, and the VH/CD ratio. These parameters reflect the metabolic and maturation state of enterocytes, with a higher VH/CD ratio indicating superior absorptive capacity [49]. In contrast to studies using higher DON doses (2.65–6 mg/kg) that reported reduced VH and VH/CD in the jejunum of piglets [48,50,51], we observed a strikingly different result, characterized by duodenal sensitivity at low-level exposure. DON exposure (0.25–2.0 mg/kg) significantly reduced duodenal VH, while jejunal and ileal morphology remained largely unaffected. This site-specific effect may be related to differential DON absorption or regional tolerance. Our finding is partially consistent with a previous report, which also observed duodenal sensitivity at 0.9 mg/kg DON, although that study additionally reported jejunal alterations [52]. Furthermore, histopathological examination provided evidence of mild intestinal lesions in piglets receiving 0.5–2.0 mg/kg DON, including observations such as epithelial cell shedding and mononuclear cell infiltration in the lamina propria. These results demonstrate that subchronic exposure to low-level DON induces subtle but significant intestinal alterations, primarily characterized by transient barrier dysfunction, morphological changes in the duodenum, and mild histopathological damage. Recent mechanistic studies further suggest that DON may impair intestinal regeneration through pathways such as the Hippo signaling cascade [53]. Moreover, gut microbiota and metabolomic interactions play a crucial role in intestinal health [54], highlighting the need to investigate whether DON-induced dysbiosis contributes to its enterotoxicity.

Regarding systemic organ health, organ indices and histomorphological characteristics remain relatively stable in healthy animals. Abnormal organ indices may reflect corresponding histomorphological alterations. The organ index, which indicates hyperplasia, congestion, atrophy, or degenerative changes, serves as a direct indicator of overall health status [55]. In this study, DON exposure did not affect the organ indices of the heart, liver, spleen, lungs or kidneys. However, hepatorenal histomorphological examination revealed mild lesions in the DON1.0 and DON2.0 groups. These results suggest that at doses of 1.0–2.0 mg/kg, DON can induce incipient pathological changes in the liver and kidneys, although the toxicity is relatively low and insufficient to alter overall organ indices.

Moreover, dietary DON exposure significantly disrupted the serum reproductive hormone profile in piglets. Significant alterations were observed even at 0.25 mg/kg. Most notably, serum FSH, LH, and PRL levels were decreased, while GnRH levels were increased throughout the trial. This imbalance in the hypothalamic–pituitary–gonadal (HPG) axis hormones suggests DON interferes with neuroendocrine regulation. The reproductive endocrine disruption caused by DON shares similarities with that of the mycotoxin ZEA. ZEA exposure has been shown to significantly suppress serum levels of key hormones, including FSH, LH, E_2_, and PROG in piglets [56]. The underlying mechanism is attributed to its interference with pituitary signaling pathways, which suppresses the synthesis and expression of FSH and LH, and its ability to disrupt the negative feedback regulation of the HPG axis, ultimately inhibiting gonadotropin secretion [57,58]. While the disruptive effects of the ZEA on piglet reproductive hormones are well-demonstrated, reports on DON are scarce. By analogy with ZEA, we speculate that DON might impair hormone synthesis and secretion, potentially through damaging hypothalamic or pituitary cells or disrupting the HPG axis. However, this remains a hypothesis, and the exact mechanisms require direct experimental verification in future studies. Concurrently, morphological analysis of the reproductive organs revealed that DON at 1.0–2.0 mg/kg induced damage, particularly severe endometrial epithelial cell shedding in the uterus. This aligns with existing evidence of DON’s reproductive toxicity, which includes inducing apoptosis in mouse endometrial stromal cells [11] and impairing porcine oocyte maturation through mechanisms such as autophagy [12]. Therefore, the integrated assessment of hormonal imbalance and organ morphology demonstrates that subchronic, low-level DON exposure compromises the reproductive system in piglets, affecting both endocrine function and organ integrity. The hormonal profile suggests mechanisms potentially analogous to those of ZEA, providing a crucial direction for future research aimed at elucidating the specific pathways of DON-induced reproductive toxicity. Critically, emerging evidence indicates that pollutants can exert synergistic reproductive toxicity [59], underscoring the need to assess the reproductive toxicity of DON in combination with other mycotoxins to better inform risk assessment.

Our findings demonstrate that even low-dose DON exposure induces subclinical multi-organ toxicity in weaned piglets, highlighting the need for both stricter mycotoxin control in feed and the development of effective mitigation strategies. Future research should explore practical dietary interventions targeting the key pathways disrupted by DON. Promising approaches could include probiotics such as *Lactobacillus delbrueckii*, which alleviate inflammation via modulation of the NF-κB signaling pathway [60]. Herbal supplements like Astragalus are known to restore intestinal barrier function and immune balance [61]. Natural antioxidants such as *Litsea cubeba* essential oil also enhance systemic antioxidant capacity in pigs [62]. Integrating such nutritional strategies with improved feed safety management could help mitigate the hidden risks posed by low-level DON contamination in pig production.

## 4. Conclusions

This study demonstrates that subchronic exposure to low doses of DON (0.25–2.0 mg/kg) induces subclinical multi-organ toxicity in weaned piglets, without significant effects on growth performance. Specifically, dietary DON exposure led to hematological alterations such as reduced PLT at doses 0.25–2.0 mg/kg. It also altered serum biochemical and lipid profiles, with increased ALT activity at 2.0 mg/kg and decreased HDL-C levels at 0.25–0.5 mg/kg. Furthermore, DON compromised systemic and tissue antioxidant defenses, evidenced by lower serum T-AOC at 0.25–2.0 mg/kg and higher MDA content in the liver, jejunum and ileum at 0.5–2.0 mg/kg. DON exposure also promoted a pro-inflammatory state in both serum, jejunum and ileum through increased TNF-α levels and decreased IL-10 levels at 0.25–2.0 mg/kg. Importantly, histopathological damage was observed in the intestine, liver, kidney, and reproductive organs at doses above 1.0 mg/kg. Moreover, even at the lowest dose of 0.25 mg/kg, DON disrupted serum reproductive hormone profiles, characterized by reduced FSH and LH levels, and elevated GnRH levels. These findings collectively reveal that low-level DON contamination poses a concealed risk to piglet health by impairing multiple physiological systems. Our results advocate for stricter control of DON in piglet feed and highlight the necessity of considering subclinical endpoints such as the specific oxidative, immune, and reproductive parameters in future risk assessments of mycotoxin exposure.

## 5. Materials and Methods

### 5.1. Chemicals, Animals, and Ethics

The DON standard used in the experiment was provided by the Institute of Agricultural Product Quality Safety and Nutrition, Jiangsu Academy of Agricultural Sciences, with a purity of >90%. Twenty female Duroc × Landrace × Yorkshire weaned piglets (28 days of age, average body weight of 7.50 ± 0.42 kg) were obtained from the Fengning Research Unit of China Agricultural University. All experimental procedures were approved by the China Agricultural University Laboratory Animal Welfare and Animal Experimental Ethical Inspection Committee (Approval No. AW40704202–1–5).

### 5.2. Experimental Design and Management

Twenty female weaned piglets were randomly divided into five groups with four replicates per group and one piglet per replicate (individually housed in stainless steel cages with a 1.4 × 0.7 × 0.6 m size). The control group (CON) was fed a corn-soybean meal basal diet, and the other four groups were fed the same basal diet contaminated with 0.25, 0.5, 1.0, and 2.0 mg/kg DON (designated as DON0.25, DON0.5, DON1.0, and DON2.0 groups, respectively) for 42 days. The basal diet was formulated according to the NRC (2012) nutritional requirements of piglets [63]. To prepare the DON-contaminated diets, the weighed DON powder was first dissolved in 10 mL of anhydrous ethanol and then pre-mixed with approximately 5 kg of the basal diet. After thorough mixing, a multifunctional feed mixer was used to blend it with an appropriate amount of basal diet to achieve the target concentrations. The ingredients composition and nutrient levels of the basal diets are presented in Supplementary Table S6. The analysis of DON and the other five major mycotoxins of zearalenone (ZEA), aflatoxin B_1_ (AFB_1_), ochratoxin A (OTA), T-2 toxin, and fumonisin B_1_ (FB_1_) in the diets was performed as described in [64], and the measured values are provided in Supplementary Table S7. During this trial, piglets were kept for six weeks with free access to feed and water. The ambient temperature was controlled at 22 to 24 °C, with good ventilation maintained. Regular immunization and deworming were conducted periodically.

### 5.3. Sample Collection and Preparation

On days 14, 28, and 42, blood samples were collected from the anterior vena cava of all piglets after a 12 h fast. For hematological analysis, a 3 mL aliquot of blood was transferred to anticoagulant tubes and stored at 4 °C. For serum preparation, another 10 mL of blood was collected without anticoagulant, allowed to clot at room temperature for 30 min, and then centrifuged at 3000× *g* for 10 min. The obtained serum was stored at −20 °C for later biochemical analysis.

At the end of the trial (day 42), all piglets were euthanized by intravenous injection of sodium pentobarbital. After exsanguination and dissection, the thoracic and abdominal cavities were immediately opened to collect tissue samples. Major organs including the heart, liver, spleen, lungs, and kidneys were collected and weighed for the calculation of organ indices. Additionally, the duodenum, jejunum, ileum, uterus, and ovary samples were also collected. The collected tissues were rinsed with 0.9% physiological saline and subsequently fixed in 4% paraformaldehyde buffer for histopathological examination and intestinal morphology analysis. Additionally, samples of liver tissue and the scraped mucosa of the duodenum, jejunum, and ileum were collected, snap-frozen in liquid nitrogen, and then stored at −80 °C until further analysis.

### 5.4. Growth Performance

The initial body weight (IBW) of each piglet was recorded at the start of the trial. On days 14, 28, and 42, fasting BWs were measured in the morning, and feed consumption was recorded to calculate average daily gain (ADG), average daily feed intake (ADFI), and the feed-to-gain ratio (F:G) for the periods of 0–14, 15–28, 29–42, and 0–42 days.

### 5.5. Hematological, Biochemical, and Lipid Metabolic Parameters

Hematological parameters, including white blood cell count (WBC), red blood cell count (RBC), hemoglobin (HGB), hematocrit (HCT), mean corpuscular volume (MCV), mean corpuscular hemoglobin (MCH), mean corpuscular hemoglobin concentration (MCHC), standard deviation of red cell distribution width (RDW-SD), coefficient of variation of red cell distribution width (RDW-CV), platelet count (PLT), platelet distribution width (PDW), and mean platelet volume (MPV), were determined using a fully automated hematology analyzer (XS-800i, Sysmex Corporation, Kobe, Japan). Serum concentrations of total protein (TP), albumin (ALB), globulin (GLB), creatinine (CREA), urea nitrogen (UN), glucose (GLU), aspartate aminotransferase (AST), alanine aminotransferase (ALT), total bilirubin (TBIL), alkaline phosphatase (ALP), cholinesterase (CHE), and lactate dehydrogenase (LDH) were measured using a fully automated biochemical analyzer (BS-420, Shenzhen Mindray Bio-Medical Electronics Co., Ltd., Shenzhen, China) with corresponding commercial assay kits (Zhongsheng Beikong Bio-technology Co., Ltd., Beijing, China). Serum lipid profiles, including total cholesterol (TC), triglyceride (TG), high-density lipoprotein cholesterol (HDL-C), and low-density lipoprotein cholesterol (LDL-C), were analyzed using enzymatic colorimetric methods on the same biochemical analyzer.

### 5.6. Antioxidant Function

The activities of glutathione peroxidase (GSH-Px), superoxide dismutase (SOD), total antioxidant capacity (T-AOC), and the concentration of malondialdehyde (MDA) in serum, liver, and the mucosa of duodenum, jejunum, and ileum were measured using commercial colorimetric assay kits (Beijing Sinouk Institute of Biological Technology, Beijing, China). All absorbance readings were performed on a microplate reader (DR-200BS, Wuxi Hiwell-Diatek Instruments Co., Ltd., Wuxi, China).

### 5.7. Immune Parameters

The levels of immunoglobulins, including immunoglobulin A (IgA), immunoglobulin G (IgG), and immunoglobulin M (IgM) in serum and liver tissue were quantified by colorimetric methods using the aforementioned fully automated biochemical analyzer. The concentrations of cytokines, including tumor necrosis factor-α (TNF-α), interleukin-1β (IL-1β), interleukin-2 (IL-2), interleukin-4 (IL-4), and interleukin-10 (IL-10) in serum, liver, and intestinal mucosa (duodenum, jejunum, ileum) were determined using enzyme-linked immunosorbent assay (ELISA) kits (Beijing Sinouk Institute of Biological Technology, Beijing, China). All ELISA analyses were conducted following the manufacturers’ instructions using the aforementioned microplate reader.

### 5.8. Intestinal Barrier Function

Serum biomarkers of intestinal barrier integrity, including lipopolysaccharide (LPS), diamine oxidase (DAO), and D-lactic acid (D-LA), were measured using commercial colorimetric kits (Beijing Sinouk Institute of Biological Technology, Beijing, China). The assays were performed according to the manufacturers’ protocols with the same microplate reader.

### 5.9. Intestinal Morphology and Histopathological Analysis

The fixed tissues (liver, kidneys, uterus, ovaries, duodenum, jejunum, and ileum) were trimmed and processed through dehydration, clearing, paraffin embedding, sectioning, and hematoxylin–eosin (HE) staining. HE-stained sections were examined under an upright microscope (Primo Star, Carl Zeiss, Oberkochen, Germany). For intestinal morphology analysis, images were captured using Image-Pro Plus 6.0 software. Villus height (VH) and crypt depth (CD) were measured on 10 intact intestinal villi per sample, and the mean values were used to calculate the villus height-to-crypt depth ratio (VH/CD). For comprehensive histopathological assessment, sections from all collected organs were evaluated for tissue integrity and pathological changes.

### 5.10. Reproductive Hormone Analysis

Serum levels of reproductive hormones, including follicle-stimulating hormone (FSH), luteinizing hormone (LH), estradiol (E_2_), gonadotropin-releasing hormone (GnRH), prolactin (PRL), and progesterone (PROG), were measured using commercial ELISA kits (Beijing Sinouk Institute of Biological Technology, Beijing, China) according to the manufacturers’ instructions, with readings taken on the same microplate reader.

### 5.11. Statistical Analysis

The experimental data were subjected to one-way analysis of variance (ANOVA) using the General Linear Model (GLM) in SAS version 9.4 [65]. Duncan’s multiple range test was employed for multiple comparisons. The results were considered statistically significant at *p* < 0.05 and highly significant at *p* < 0.01.

## Figures and Tables

**Table 1:** Effects of dietary DON exposure on growth performance of piglets.

Items	Treatments					SEM	*p*-Values
	CON	DON0.25	DON0.5	DON1.0	DON2.0		
Initial BW (kg)	7.44	7.49	7.56	7.54	7.45	0.42	0.999
14 d BW (kg)	10.60	10.66	10.56	11.15	11.10	0.84	0.975
28 d BW (kg)	19.13	19.61	18.75	19.86	18.34	1.77	0.972
42 d BW (kg)	28.71	29.96	27.86	30.45	26.93	2.38	0.823
0–14 d							
ADG (kg)	0.23	0.23	0.21	0.26	0.26	0.05	0.958
ADFI (kg)	0.39	0.41	0.37	0.46	0.40	0.07	0.911
F:G	1.74	1.80	1.72	1.80	1.73	0.09	0.943
15–28 d							
ADG (kg)	0.61	0.64	0.58	0.62	0.52	0.07	0.802
ADFI (kg)	0.96	1.07	0.96	0.98	0.83	0.11	0.665
F:G	1.57	1.70	1.68	1.60	1.61	0.09	0.811
29–42 d							
ADG (kg)	0.68	0.74	0.65	0.76	0.61	0.06	0.397
ADFI (kg)	1.32	1.40	1.21	1.44	1.11	0.10	0.184
F:G	1.93	1.90	1.86	1.91	1.85	0.07	0.903
0–42 d							
ADG (kg)	0.51	0.54	0.48	0.55	0.46	0.05	0.805
ADFI (kg)	0.89	0.96	0.85	0.96	0.78	0.09	0.562
F:G	1.76	1.80	1.75	1.76	1.71	0.04	0.669

Note: BW, Body weight; ADG, Average daily gain; ADFI, Average daily feed intake; F:G, Feed-to-gain ratio. SEM, standard error of the mean. *n* = 4.

**Table 2:** Effects of dietary DON exposure on hematological and biochemical parameters of piglets.

Items	Treatments					SEM	*p*-Values
	CON	DON0.25	DON0.5	DON1.0	DON2.0		
Hematological parameters							
28 d							
PLT (10^9^/L)	520.00 ^A^	262.00 ^B^	312.25 ^B^	358.50 ^B^	304.25 ^B^	41.28	0.005
42 d							
PDW (fL)	14.18 ^A^	10.58 ^B^	9.98 ^B^	10.38 ^B^	15.75 ^A^	1.00	0.003
MPV (fL)	11.48 ^A^	9.80 ^B^	9.25 ^B^	10.15 ^B^	11.55 ^A^	0.43	0.006
Biochemical parameters							
28 d							
AST (U/L)	54.92 ^B^	66.85 ^B^	117.32 ^A^	54.92 ^B^	49.72 ^B^	7.78	<0.001
42 d							
AST (U/L)	39.61 ^b^	48.65 ^b^	74.64 ^a^	59.07 ^ab^	49.71 ^b^	7.59	0.049
ALT (U/L)	48.51 ^b^	50.52 ^ab^	58.27 ^ab^	60.77 ^ab^	70.68 ^a^	4.69	0.031

Note: PLT, platelet count; PDW, platelet distribution width; MPV, mean platelet volume; AST, aspartate aminotransferase; ALT, alanine aminotransferase. SEM, standard error of the mean. *n* = 4. ^A,B^, different letters mean a statistical difference (*p* < 0.01). ^a,b^, different letters mean a statistical difference (*p* < 0.05).

**Table 3:** Effects of dietary DON exposure on serum lipid profiles of piglets (mmol/L).

Items	Treatments					SEM	*p*-Values
	CON	DON0.25	DON0.5	DON1.0	DON2.0		
14 d							
TC	2.09	1.87	1.92	1.59	1.64	0.29	0.727
TG	0.33 ^bc^	0.48 ^a^	0.30 ^c^	0.44 ^ab^	0.30 ^c^	0.04	0.027
HDL-C	0.75	0.64	0.59	0.62	0.59	0.04	0.082
LDL-C	1.60	1.58	1.74	1.43	1.54	0.21	0.873
28 d							
TC	1.65	1.42	1.52	1.55	1.97	0.17	0.228
TG	0.39	0.39	0.39	0.32	0.30	0.05	0.581
HDL-C	0.71 ^a^	0.57 ^bc^	0.50 ^c^	0.63 ^abc^	0.66 ^ab^	0.04	0.036
LDL-C	1.37 ^b^	1.42 ^b^	1.61 ^ab^	1.56 ^ab^	1.75 ^a^	0.09	0.046
42 d							
TC	1.88	1.76	1.50	1.69	1.66	0.15	0.510
TG	0.30	0.26	0.43	0.25	0.30	0.04	0.083
HDL-C	0.71 ^a^	0.69 ^a^	0.60 ^b^	0.65 ^ab^	0.66 ^ab^	0.02	0.018
LDL-C	1.64	1.60	1.49	1.60	1.56	0.11	0.917

Note: TC, total cholesterol; TG, triglyceride; HDL-C, high-density lipoprotein cholesterol; LDL-C, low-density lipoprotein cholesterol. SEM, standard error of the mean. *n* = 4. ^a–c^, different letters mean a statistical difference (*p* < 0.05).

**Table 4:** Effects of dietary DON exposure on serum antioxidant function of piglets.

Items	Treatments					SEM	*p*-Values
	CON	DON0.25	DON0.5	DON1.0	DON2.0		
14 d							
GSH-Px (U/mL)	163.15	166.93	165.27	161.32	164.03	1.62	0.197
SOD (U/mL)	69.41	61.74	66.21	50.68	63.16	7.36	0.472
T-AOC (U/mL)	8.98 ^A^	6.33 ^B^	6.89 ^B^	5.61 ^B^	6.95 ^B^	0.56	0.009
MDA (nmol/mL)	4.71 ^b^	4.79 ^b^	4.49 ^b^	5.66 ^a^	4.50 ^b^	0.22	0.012
28 d							
GSH-Px (U/mL)	169.35	162.85	165.84	164.49	164.97	1.42	0.058
SOD (U/mL)	88.34	88.50	85.63	85.76	85.97	3.36	0.943
T-AOC (U/mL)	9.55 ^A^	8.10 ^BC^	8.69 ^AB^	7.62 ^C^	7.48 ^C^	0.34	0.004
MDA (nmol/mL)	3.55	3.53	3.61	3.57	3.59	0.42	1.000
42 d							
GSH-Px (U/mL)	172.25	165.56	158.31	162.33	162.35	3.37	0.098
SOD (U/mL)	93.71	93.44	90.21	87.73	88.04	2.31	0.244
T-AOC (U/mL)	10.69 ^A^	9.04 ^BC^	9.63 ^B^	8.66 ^C^	8.58 ^C^	0.28	0.001
MDA (nmol/mL)	3.04	3.09	3.15	3.32	3.25	0.32	0.969

Note: GSH-Px, glutathione peroxidase; SOD, superoxide dismutase; T-AOC, total antioxidant capacity; MDA, malondialdehyde. SEM, standard error of the mean. *n* = 4. ^A–C^, different letters mean a statistical difference (*p* < 0.01). ^a,b^, different letters mean a statistical difference (*p* < 0.05).

**Table 5:** Effects of 42-day dietary DON exposure on organ indices of piglets (g/kg).

Items	Treatments					SEM	*p*-Values
	CON	DON0.25	DON0.5	DON1.0	DON2.0		
Heart index	5.16	5.14	5.20	4.63	4.50	0.32	0.401
Liver index	24.35	23.65	24.04	23.58	22.53	1.01	0.758
Spleen index	1.90	1.90	1.99	1.94	2.04	0.16	0.962
Lung index	9.60	9.41	9.97	10.11	9.93	0.55	0.885
Kidney index	5.11	4.62	5.28	4.99	4.54	0.21	0.111

Note: SEM, standard error of the mean. *n* = 4.

## Data Availability

The original contributions presented in this study are included in the article/Supplementary Materials. Further inquiries can be directed to the corresponding author.

## Supplementary Materials

The following supporting information can be downloaded at: https://www.mdpi.com/article/10.3390/toxins18020075/s1, Table S1: Effects of dietary DON exposure on hematological parameters of piglets; Table S2: Effects of dietary DON exposure on serum biochemical parameters of piglets; Table S3: Effects of dietary DON exposure on antioxidant function in the liver and intestine of piglets; Table S4: Effects of dietary DON exposure on serum immunoglobulin levels of piglets; Table S5: Effects of dietary DON exposure on intestinal mucosal immune cytokine levels of piglets; Table S6: Ingredients composition and nutrient levels of the basal diet (%, as-fed basis); Table S7: The determination of mycotoxins in treatment diets (µg/kg); Figure S1: Effects of dietary DON exposure on liver immunoglobulin levels of piglets; Figure S2: Effects of dietary DON exposure on liver immune cytokine levels of piglets.

## References

[1] Li Y., Gao H., Wang R., Xu Q. (2023). Deoxynivalenol in food and feed: Recent advances in decontamination strategies. Front. Microbiol..

[2] Pinto A.C.S.M., De Pierri C.R., Evangelista A.G., Gomes A.S.d.L.P.B., Luciano F.B. (2022). Deoxynivalenol: Toxicology, degradation by bacteria, and phylogenetic analysis. Toxins.

[3] Mishra S., Srivastava S., Dewangan J., Divakar A., Kumar Rath S. (2020). Global occurrence of deoxynivalenol in food commodities and exposure risk assessment in humans in the last decade: A survey. Crit. Rev. Food Sci. Nutr..

[4] Gruber-Dorninger C., Jenkins T., Schatzmayr G. (2019). Global mycotoxin occurrence in feed: A ten-year survey. Toxins.

[5] Biscoto G.L., Salvato L.A., Alvarenga E.R., Dias R.R.S., Pinheiro G.R.G., Rodrigues M.P., Pinto P.N., Freitas R.P., Keller K.M. (2022). Mycotoxins in cattle feed and feed ingredients in Brazil: A five-year survey. Toxins.

[6] Tu Y., Liu S., Cai P., Shan T. (2023). Global distribution, toxicity to humans and animals, biodegradation, and nutritional mitigation of deoxynivalenol: A review. Compr. Rev. Food Sci. Food Saf..

[7] Sobrova P., Adam V., Vasatkova A., Beklova M., Zeman L., Kizek R. (2010). Deoxynivalenol and its toxicity. Interdiscip. Toxicol..

[8] Kang T.H., Kang K.S., Lee S.I. (2022). Deoxynivalenol induces apoptosis via FOXO3a-signaling pathway in small-intestinal cells in pig. Toxics.

[9] Wang X., Chen X., Cao L., Zhu L., Zhang Y., Chu X., Zhu D., Rahman S.U., Peng C., Feng S. (2020). Mechanism of deoxynivalenol-induced neurotoxicity in weaned piglets is linked to lipid peroxidation, dampened neurotransmitter levels, and interference with calcium signaling. Ecotoxicol. Environ. Saf..

[10] Yang L.N., Xu S., Tang M., Zhou X., Liao Y., Nussler A.K., Liu L., Yang W. (2023). The circadian rhythm gene bmal1 ameliorates acute deoxynivalenol-induced liver damage. Arch. Toxicol..

[11] Dai Y., Xie H., Xu Y. (2017). Evaluation of deoxynivalenol-induced toxic effects on mouse endometrial stromal cells: Cell apoptosis and cell cycle. Biochem. Biophys. Res. Commun..

[12] Han J., Wang Q.C., Zhu C.C., Liu J., Zhang Y., Cui X.S., Kim N.H., Sun S.C. (2016). Deoxynivalenol exposure induces autophagy/apoptosis and epigenetic modification changes during porcine oocyte maturation. Toxicol. Appl. Pharmacol..

[13] Ghareeb K., Awad W.A., Soodoi C., Sasgary S., Strasser A., Bohm J. (2013). Effects of feed contaminant deoxynivalenol on plasma cytokines and mRNA expression of immune genes in the intestine of broiler chickens. PLoS ONE.

[14] Hou S., Cheng Y., Wang Z., Xia L., Wang J., Wang H., Sun J., Ma J., Yan Y. (2023). DON entry into the nucleus induces DNA damage, apoptosis and cycle arrest in GES-1 cells. Food Chem. Toxicol..

[15] Yu M., Peng Z., Liao Y., Wang L., Li D., Qin C., Hu J., Wang Z., Cai M., Cai Q. (2019). Deoxynivalenol-induced oxidative stress and nrf2 translocation in maternal liver on gestation day 12.5 d and 18.5 d. Toxicon.

[16] Liu M., Zhang L., Mo Y., Li J., Yang J., Wang J., Karrow N.A., Wu H., Sun L. (2023). Ferroptosis is involved in deoxynivalenol-induced intestinal damage in pigs. J. Anim. Sci. Biotechnol..

[17] Liao P., Li Y., Li M., Chen X., Yuan D., Tang M., Xu K. (2020). Baicalin alleviates deoxynivalenol-induced intestinal inflammation and oxidative stress damage by inhibiting NF-kappab and increasing mTOR signaling pathways in piglets. Food Chem. Toxicol..

[18] Li J., Bai Y., Ma K., Ren Z., Li J., Zhang J., Shan A. (2022). Dihydroartemisinin alleviates deoxynivalenol induced liver apoptosis and inflammation in piglets. Ecotoxicol. Environ. Saf..

[19] Payros D., Alassane-Kpembi I., Pierron A., Loiseau N., Pinton P., Oswald I.P. (2016). Toxicology of deoxynivalenol and its acetylated and modified forms. Arch. Toxicol..

[20] Dänicke S., Valenta H., Döll S. (2004). On the toxicokinetics and the metabolism of deoxynivalenol (DON) in the pig. Arch. Anim. Nutr..

[21] Prelusky D.B., Hartin K.E., Trenholm H.L., Miller J.D. (1988). Pharmacokinetic fate of 14c-labeled deoxynivalenol in swine. Fundam. Appl. Toxicol..

[22] Prelusky D.B., Veira D.M., Trenholm H.L. (1985). Plasma pharmacokinetics of the mycotoxin deoxynivalenol following oral and intravenous administration to sheep. J. Environ. Sci. Health B.

[23] Osselaere A., Devreese M., Goossens J., Vandenbroucke V., De Baere S., De Backer P., Croubels S. (2013). Toxicokinetic study and absolute oral bioavailability of deoxynivalenol, T-2 toxin and zearalenone in broiler chickens. Food Chem. Toxicol..

[24] Rohweder D., Kersten S., Valenta H., Sondermann S., Schollenberger M., Drochner W., Dänicke S. (2013). Bioavailability of the Fusarium toxin deoxynivalenol (DON) from wheat straw and chaff in pigs. Arch. Anim. Nutr..

[25] Prelusky D.B., Trenholm H.L., Lawrence G.A., Scott P.M. (1984). Nontransmission of deoxynivalenol (vomitoxin) to milk following oral administration to dairy cows. J. Environ. Sci. Health B.

[26] Frey J.C., Pell A.N., Berthiaume R., Lapierre H., Lee S., Ha J.K., Mendell J.E., Angert E.R. (2010). Comparative studies of microbial populations in the rumen, duodenum, ileum and faeces of lactating dairy cows. J. Appl. Microbiol..

[27] Goyarts T., Danicke S., Valenta H., Ueberschar K.H. (2007). Carry-over of fusarium toxins (deoxynivalenol and zearalenone) from naturally contaminated wheat to pigs. Food Addit. Contam..

[28] Goyarts T., Danicke S., Brussow K.P., Valenta H., Ueberschar K.H., Tiemann U. (2007). On the transfer of the fusarium toxins deoxynivalenol (DON) and zearalenone (ZON) from sows to their fetuses during days 35–70 of gestation. Toxicol. Lett..

[29] European Commission (EC) (2006). Commission Recommendation 576/2006/EC of 17 August 2006 on the presence of deoxynivalenol, zearalenone, ochratoxin A, T-2 and HT-2 and fumonisins in products intended for animal feeding. Off. J. Eur. Union.

[30] U.S. Food and Drug Administration (2020). Guidance for Industry and FDA: Advisory Levels for Deoxynivalenol (DON) in Finished Wheat Products for Human Consumption and Grains and Grain By-Products Used for Animal Feed.

[31] (2017). Hygienical Standard for Feeds.

[32] Holanda D.M., Kim Y.I., Parnsen W., Kim S.W. (2021). Phytobiotics with adsorbent to mitigate toxicity of multiple mycotoxins on health and growth of pigs. Toxins.

[33] Pratt D.S., Kaplan M.M. (2000). Evaluation of abnormal liver-enzyme results in asymptomatic patients. N. Engl. J. Med..

[34] Wu L., Liao P., He L., Ren W., Yin J., Duan J., Li T. (2015). Growth performance, serum biochemical profile, jejunal morphology, and the expression of nutrients transporter genes in deoxynivalenol (DON)-challenged growing pigs. BMC Vet. Res..

[35] Xu X., Chang J., Wang P., Liu C., Liu M., Zhou T., Yin Q., Yan G. (2022). Glycyrrhizic acid and compound probiotics supplementation alters the intestinal transcriptome and microbiome of weaned piglets exposed to deoxynivalenol. Toxins.

[36] Ali O., Mezes M., Balogh K., Kovacs M., Szabo A. (2021). The effects of mixed fusarium mycotoxins at EU-permitted feed levels on weaned piglets’ tissue lipids. Toxins.

[37] Mishra S., Dwivedi P.D., Pandey H.P., Das M. (2014). Role of oxidative stress in deoxynivalenol induced toxicity. Food Chem. Toxicol..

[38] Wu M., Xiao H., Ren W., Yin J., Tan B., Liu G., Li L., Nyachoti C.M., Xiong X., Wu G. (2014). Therapeutic effects of glutamic acid in piglets challenged with deoxynivalenol. PLoS ONE.

[39] Pestka J.J. (2008). Mechanisms of deoxynivalenol-induced gene expression and apoptosis. Food Addit. Contam. Part A.

[40] Rocha O., Ansari K., Doohan F.M. (2005). Effects of trichothecene mycotoxins on eukaryotic cells: A review. Food Addit. Contam..

[41] Ren Z.H., Deng H.D., Wang Y.C., Deng J.L., Zuo Z.C., Wang Y., Peng X., Cui H.M., Fang J., Yu S.M. (2016). The fusarium toxin zearalenone and deoxynivalenol affect murine splenic antioxidant functions, interferon levels, and T-cell subsets. Environ. Toxicol. Pharmacol..

[42] Liao Y., Peng Z., Chen L., Nussler A.K., Liu L., Yang W. (2018). Deoxynivalenol, gut microbiota and immunotoxicity: A potential approach?. Food Chem. Toxicol..

[43] Reddy K.E., Song J., Lee H.J., Kim M., Kim D.W., Jung H.J., Kim B., Lee Y., Yu D., Kim D.W. (2018). Effects of high levels of deoxynivalenol and zearalenone on growth performance, and hematological and immunological parameters in pigs. Toxins.

[44] Qiu Y., Nie X., Yang J., Wang L., Zhu C., Yang X., Jiang Z. (2022). Effect of resveratrol supplementation on intestinal oxidative stress, immunity and gut microbiota in weaned piglets challenged with deoxynivalenol. Antioxidants.

[45] Xu X., Chang J., Wang P., Liu C., Liu M., Zhou T., Yin Q., Yan G. (2023). Combination of glycyrrhizic acid and compound probiotics alleviates deoxynivalenol-induced damage to weaned piglets. Ecotoxicol. Environ. Saf..

[46] Yacoub M.R., Ramirez G.A., Berti A., Mercurio G., Breda D., Saporiti N., Burastero S., Dagna L., Colombo G. (2018). Diamine oxidase supplementation in chronic spontaneous urticaria: A randomized, double-blind placebo-controlled study. Int. Arch. Allergy Immunol..

[47] Verhoeven P.O., Gagnaire J., Haddar C.H., Grattard F., Thibaudin D., Afiani A., Cazorla C., Carricajo A., Mariat C., Alamartine E. (2016). Identifying hemodialysis patients with the highest risk of *Staphylococcus aureus* endogenous infection through a simple nasal sampling algorithm. Medicine.

[48] Hong Q., Li X., Lin Q., Shen Z., Feng J., Hu C. (2022). Resveratrol improves intestinal morphology and anti-oxidation ability in deoxynivalenol-challenged piglets. Animals.

[49] Fan M.Z., Stoll B., Jiang R., Burrin D.G. (2001). Enterocyte digestive enzyme activity along the crypt-villus and longitudinal axes in the neonatal pig small intestine. J. Anim. Sci..

[50] Wu L., Liao P., He L., Feng Z., Ren W., Yin J., Duan J., Li T., Yin Y. (2015). Dietary L-arginine supplementation protects weanling pigs from deoxynivalenol-induced toxicity. Toxins.

[51] Ji X., Ding H., Zhou F., Zhang F., Wu D. (2025). Taurine ameliorates deoxynivalenol-induced intestinal injury in piglets: Restoration of mitochondrial function linked to the PGC1alpha-NRF1/2 axis. Ecotoxicol. Environ. Saf..

[52] Alizadeh A., Braber S., Akbari P., Garssen J., Fink-Gremmels J. (2015). Deoxynivalenol impairs weight gain and affects markers of gut health after low-dose, short-term exposure of growing pigs. Toxins.

[53] Liao P. (2025). Deoxynivalenol regulates intestinal and stem cell regeneration via the Hippo pathway and clinical intervention strategies. Toxicon.

[54] Cheng X., Tan Y., Li H., Huang J., Zhao D., Zhang Z., Yi M., Zhu L., Hui S., Yang J. (2022). Fecal 16S rRNA sequencing and multi-compartment metabolomics revealed gut microbiota and metabolites interactions in APP/PS1 mice. Comput. Biol. Med..

[55] He W., Gu A., Wang D. (2023). Four-week repeated exposure to tire-derived 6-PPD quinone causes multiple organ injury in male BALB/c mice. Sci. Total Environ..

[56] Fu G., Wang L., Li L., Liu J., Liu S., Zhao X. (2018). *Bacillus licheniformis* CK1 alleviates the toxic effects of zearalenone in feed on weaned female Tibetan piglets. J. Anim. Sci..

[57] Zheng W., Feng N., Wang Y., Noll L., Xu S., Liu X., Lu N., Zou H., Gu J., Yuan Y. (2019). Effects of zearalenone and its derivatives on the synthesis and secretion of mammalian sex steroid hormones: A review. Food Chem. Toxicol..

[58] He J., Wei C., Li Y., Liu Y., Wang Y., Pan J., Liu J., Wu Y., Cui S. (2018). Zearalenone and alpha-zearalenol inhibit the synthesis and secretion of pig follicle stimulating hormone via the non-classical estrogen membrane receptor GPR30. Mol. Cell Endocrinol..

[59] Zhang J., Zhang Y., Xia X., Ma C., Zhang Q., Li Y., Zhang Q., Wen W., Yang Z. (2025). Promoting effects of lipophilic pollutants on the reproductive toxicity of proteinophilic pollutants to *Daphnia magna* under chronic exposure. Environ. Pollut..

[60] Zhong S., Sun Z., Tian Q., Wen W., Chen F., Huang X., Li Y. (2024). *Lactobacillus delbrueckii* alleviates lipopolysaccharide-induced muscle inflammation and atrophy in weaned piglets associated with inhibition of endoplasmic reticulum stress and protein degradation. FASEB J..

[61] Su M., Tang T., Tang W., Long Y., Wang L., Liu M. (2023). Astragalus improves intestinal barrier function and immunity by acting on intestinal microbiota to treat T2DM: A research review. Front. Immunol..

[62] Chen F., Wang Y., Wang K., Chen J., Jin K., Peng K., Chen X., Liu Z., Ouyang J., Wang Y. (2023). Effects of *Litsea cubeba* essential oil on growth performance, blood antioxidation, immune function, apparent digestibility of nutrients, and fecal microflora of pigs. Front. Pharmacol..

[63] National Research Council (2012). Nutrient Requirements of Swine.

[64] Liu Y., Jin Y., Guo Q., Wang X., Luo S., Yang W., Li J., Chen Y. (2022). Immunoaffinity cleanup and isotope dilution-based liquid chromatography tandem mass spectrometry for the determination of six major mycotoxins in feed and feedstuff. Toxins.

[65] SAS Institute Inc (2013). SAS/STAT 9.4 User’s Guide.

