# Supplementary material for: Low-Dose Deoxynivalenol Induces Subclinical Multi-Organ Toxicity in Weaned Piglets
Source: Toxins (Basel). 2026 Feb 2;18(2):75. doi: 10.3390/toxins18020075 (PMC12945029; doi:10.3390/toxins18020075)
Supplement: Supplementary file 1 [file toxins-18-00075-s001.zip › toxins-4095006-supplementary.pdf]

Supplementary Materials: Low-dose Deoxynivalenol induces subclinical multi-organ toxicity in weaned piglets

Ying Liu, Sunlin Luo, Xinchun Zou, Wenjun He, Ruiqi Tan, Yongpeng Jin, Gaoyi Liu, Qiaomin Duan, Wenjun Yang, and Yiqiang Chen

Table S1. Effects of dietary DON exposure on hematological parameters of piglets.

Items	Treatments					SEM	*p*-Values
	CON	DON0.25	DON0.5	DON1.0	DON2.0		
**14 d**							
WBC (10^9/L)	14.07	12.14	16.43	14.15	16.62	2.49	0.694
RBC (10^{12}/L)	6.02	6.08	5.93	5.65	6.17	0.26	0.693
HGB (g/L)	108.25	109.25	110.50	103.50	114.25	5.16	0.689
HCT (%)	41.90	41.35	42.85	39.73	44.70	1.54	0.273
MCV (fL)	69.73	68.35	72.35	70.30	72.75	2.46	0.695
MCH (pg)	18.00	18.08	18.58	18.30	18.55	0.67	0.957
MCHC (g/L)	258.50	264.25	256.75	260.50	255.75	5.08	0.776
RDW-SD (fL)	58.18	55.85	61.83	52.63	62.28	2.54	0.081
RDW-CV (%)	25.10	24.85	25.63	22.35	25.68	1.05	0.204
PLT (10^9/L)	342.00	290.00	356.00	325.50	408.50	63.10	0.754
PDW (fL)	20.80	20.80	20.95	21.18	20.70	0.83	0.995
MPV (fL)	13.43	13.15	13.50	13.48	13.43	0.21	0.784
28 d							
WBC (10^9/L)	11.92	12.10	15.08	14.67	16.72	2.25	0.526
RBC (10^{12}/L)	4.76	4.82	4.39	4.77	4.85	0.27	0.740
HGB (g/L)	79.75	84.00	73.50	82.00	83.50	3.76	0.318
HCT (%)	25.00	27.13	23.70	25.78	26.78	1.30	0.378
MCV (fL)	52.80	56.35	54.38	54.08	55.45	2.11	0.795
MCH (pg)	16.88	17.45	16.83	17.20	17.30	0.59	0.926
MCHC (g/L)	319.25	310.00	310.00	318.25	311.75	4.03	0.326
RDW-SD (fL)	38.90	41.95	40.53	35.95	40.73	1.69	0.167
RDW-CV (%)	21.80	22.13	22.50	19.98	21.93	1.05	0.507
PLT (10^9/L)	520.00^{A}	262.00^{B}	312.25^{B}	358.50^{B}	304.25^{B}	41.28	0.005
PDW (fL)	19.45	24.60	24.73	21.75	20.43	1.75	0.167
MPV (fL)	12.65	13.90	14.32	13.58	12.79	0.47	0.109
42 d							
WBC (10^9/L)	15.02	13.45	15.03	12.89	14.89	1.24	0.626
RBC (10^{12}/L)	5.37	5.37	5.15	5.37	5.72	0.27	0.687
HGB (g/L)	89.50	92.25	88.25	93.25	98.00	3.96	0.478
HCT (%)	28.08	29.85	28.45	28.83	30.40	1.24	0.654
MCV (fL)	52.53	55.68	55.43	53.70	53.50	1.94	0.750
MCH (pg)	16.73	17.20	17.18	17.38	17.23	0.54	0.933
MCHC (g/L)	319.00	309.25	310.00	324.00	322.00	4.42	0.097
RDW-SD (fL)	35.13	38.83	36.75	36.80	37.30	1.04	0.218
RDW-CV (%)	20.53	21.18	20.28	21.03	21.40	0.83	0.865
PLT (10^9/L)	311.00	271.75	240.25	287.00	234.50	55.33	0.850
PDW (fL)	14.18^{A}	10.58^{B}	9.98^{B}	10.38^{B}	15.75^{A}	1.00	0.003
MPV (fL)	11.48^{A}	9.80^{B}	9.25^{B}	10.15^{B}	11.55^{A}	0.43	0.006

Note: WBC, white blood cell count; RBC, red blood cell count; HGB, hemoglobin; HCT, hematocrit; MCV, mean corpuscular volume; MCH, mean corpuscular hemoglobin; MCHC, mean corpuscular hemoglobin concentration; RDW-SD, standard deviation of red cell distribution width; RDW-CV, coefficient of variation of red cell distribution width; PLT, platelet count; PDW, platelet distribution width; MPV, mean platelet volume. SEM, standard error of the mean. $n = 4$. $^{A–B}$, different letters mean a statistical difference ($p < 0.01$)

Table S2. Effects of dietary DON exposure on serum biochemical parameters of piglets.

Items	Treatments					SEM	*p*-Values
	CON	DON0.25	DON0.5	DON1.0	DON2.0		
**14 d**							
TP (g/L)	53.91	52.12	54.87	47.04	53.33	2.44	0.226
ALB (g/L)	26.22	22.62	24.17	21.93	22.76	1.59	0.371
GLB (g/L)	27.68	29.50	30.71	25.10	30.57	2.93	0.639
CREA (μmol/L)	71.12	68.59	68.12	70.08	81.03	4.66	0.316
UN (mg/dL)	7.44	12.83	12.72	10.72	10.42	1.28	0.057
GLU (mmol/L)	4.38	3.21	3.64	4.04	4.71	0.54	0.359
AST (U/L)	53.18	83.40	71.28	87.95	78.82	11.91	0.311
ALT (U/L)	66.30	72.17	54.91	64.13	52.88	5.62	0.138
TBIL (μmol/L)	6.69	4.81	7.44	6.15	7.55	0.80	0.151
ALP (U/L)	233.03	184.05	158.11	217.24	239.25	34.59	0.443
CHE (U/L)	264.44	353.55	279.71	321.56	378.24	35.70	0.179
LDH (U/L)	638.23	550.27	559.72	471.12	606.34	110.56	0.854
28 d							
TP (g/L)	51.49	55.75	59.06	51.93	53.16	2.93	0.372
ALB (g/L)	21.43	23.10	20.47	20.38	22.33	1.53	0.674
GLB (g/L)	30.06	32.65	38.59	31.55	30.83	3.61	0.492
CREA (μmol/L)	74.34	78.74	72.68	78.23	79.85	3.89	0.649
UN (mg/dL)	11.63	10.67	10.53	11.14	10.37	1.58	0.979
GLU (mmol/L)	3.53	4.01	3.15	3.88	4.12	0.44	0.533
AST (U/L)	54.92^{B}	66.85^{B}	117.32^{A}	54.92^{B}	49.72^{B}	7.78	< 0.001
ALT (U/L)	50.12	64.30	66.06	59.54	54.47	6.36	0.394
TBIL(μmol/L)	5.57	6.30	6.28	6.40	8.39	0.77	0.168
ALP (U/L)	215.10	199.82	190.80	164.36	251.29	31.51	0.420
CHE (U/L)	271.04	279.43	312.20	217.73	311.65	42.72	0.532
LDH (U/L)	491.84	600.38	838.11	477.83	520.21	138.32	0.368
42 d							
TP (g/L)	55.80	59.46	60.20	58.27	54.82	1.39	0.066
ALB (g/L)	26.18	26.06	26.14	27.17	22.97	1.37	0.297
GLB (g/L)	29.63	33.41	34.06	31.12	31.85	1.62	0.347
CREA (μmol/L)	87.38	89.94	86.68	89.58	91.49	3.03	0.791
UN (mg/dL)	10.91	11.28	11.88	13.13	10.42	1.37	0.680
GLU(mmol/L)	4.71	4.78	4.38	5.43	5.54	0.42	0.271
AST (U/L)	39.61^{b}	48.65^{b}	74.64^{a}	59.07^{ab}	49.71^{b}	7.59	0.049
ALT (U/L)	48.51^{b}	50.52^{ab}	58.27^{ab}	60.77^{ab}	70.68^{a}	4.69	0.031
TBIL(μmol/L)	5.88	7.11	5.48	7.29	5.59	0.69	0.240
ALP (U/L)	190.46	183.57	192.46	180.30	204.98	21.50	0.936
CHE (U/L)	178.58	221.08	243.51	243.73	239.25	33.49	0.614
LDH (U/L)	541.61	509.56	537.49	419.62	518.04	90.83	0.874

Note: TP, total protein; ALB, albumin; GLB, globulin; CREA, creatinine; UN, urea nitrogen; GLU, glucose; AST, aspartate aminotransferase; ALT, alanine aminotransferase; TBIL, total bilirubin; ALP, alkaline phosphatase; CHE, cholinesterase; LDH, lactate dehydrogenase. SEM, standard error of the

mean. n = 4. $^{A–B}$, different letters mean a statistical difference ($p < 0.01$). $^{a–b}$, different letters mean a statistical difference ($p < 0.05$).

Table S3. Effects of dietary DON exposure on antioxidant function in the liver and intestine of piglets.

Items	Treatments					SEM	p-Values
	CON	DON0.25	DON0.5	DON1.0	DON2.0		
**Liver**							
GSH-Px (U/mg prot)	18.30^{a}	16.45^{ab}	15.66^{b}	15.49^{b}	15.64^{b}	0.64	0.036
SOD (U/mg prot)	7.75	7.28	6.86	6.97	7.24	0.35	0.447
T-AOC (U/mg prot)	1.88	1.53	1.28	1.42	1.44	0.14	0.078
MDA (nmol/mg prot)	1.38^{C}	1.71^{B}	1.99^{A}	1.91^{AB}	1.79^{AB}	0.09	0.002
Duodenal mucosa							
GSH-Px (U/mg prot)	15.04	14.78	14.77	14.75	14.77	0.74	0.998
SOD (U/mg prot)	6.54	6.05	5.78	6.03	5.84	0.43	0.753
T-AOC (U/mg prot)	1.63	1.48	1.44	1.40	1.44	0.08	0.351
MDA (nmol/mg prot)	2.27	2.48	2.71	2.70	2.52	0.31	0.841
Jejunal mucosa							
GSH-Px (U/mg prot)	17.74^{a}	17.39^{ab}	12.96^{c}	14.86^{abc}	14.40^{bc}	0.95	0.013
SOD (U/mg prot)	5.98	5.64	5.27	5.41	5.85	0.33	0.547
T-AOC (U/mg prot)	1.45^{a}	1.44^{a}	1.13^{b}	1.25^{ab}	1.08^{b}	0.09	0.031
MDA (nmol/mg prot)	1.83^{b}	2.09^{ab}	2.45^{a}	2.29^{a}	2.20^{a}	0.11	0.017
Ileal mucosa							
GSH-Px (U/mg prot)	16.46	14.60	13.74	15.04	15.30	0.73	0.175
SOD (U/mg prot)	6.07^{a}	5.96^{a}	4.56^{b}	5.36^{ab}	5.75^{a}	0.30	0.020
T-AOC (U/mg prot)	1.45	1.43	1.26	1.27	1.39	0.08	0.378
MDA (nmol/mg prot)	1.80^{c}	1.95^{bc}	2.40^{a}	2.34^{a}	2.24^{ab}	0.12	0.012

Note: GSH-Px, glutathione peroxidase; SOD, superoxide dismutase; T-AOC, total antioxidant capacity; MDA, malondialdehyde. SEM, standard error of the mean. n = 4. $^{A–C}$, different letters mean a statistical difference ($p < 0.01$). $^{a–c}$, different letters mean a statistical difference ($p < 0.05$).

Table S4. Effects of dietary DON exposure on serum immunoglobulin levels of piglets (g/L).

Items	Treatments					SEM	p-Values
	CON	DON0.25	DON0.5	DON1.0	DON2.0		
**14 d**							
IgA	1.28	1.28	1.36	1.11	1.31	0.11	0.608
IgG	14.76	18.44	18.45	12.06	18.30	2.12	0.169
IgM	2.05	2.16	2.36	1.96	2.23	0.18	0.548
28 d							
IgA	1.24	1.49	1.51	1.36	1.31	0.10	0.328
IgG	18.50	20.20	21.22	19.82	19.39	0.77	0.202
IgM	2.26	2.85	2.65	2.55	2.39	0.21	0.368

42 d							
IgA	1.30	1.49	1.53	1.37	1.41	0.06	0.092
IgG	18.52	20.73	21.25	19.51	20.06	0.85	0.238
IgM	2.61	2.70	2.83	2.49	2.63	0.11	0.315

Note: IgA, immunoglobulin A; IgG, immunoglobulin G; IgM, immunoglobulin M. SEM, standard error of the mean. $n = 4$.

Table S5. Effects of dietary DON exposure on intestinal mucosal immune cytokine levels of piglets (pg/mg prot).

Items	Treatments					SEM	p-Values
	CON	DON0.25	DON0.5	DON1.0	DON2.0		
**Duodenal mucosa**							
TNF-α	4.94	5.55	5.74	5.29	5.22	0.44	0.746
IL-1β	2.27	2.50	2.59	2.54	2.46	0.17	0.716
IL-2	22.41	24.22	24.88	24.46	24.24	0.82	0.295
IL-4	1.58	1.42	1.36	1.51	1.40	0.11	0.659
IL-10	2.47	2.40	2.34	2.35	2.45	0.12	0.888
Jejunal mucosa							
TNF-α	4.43^{C}	4.53^{C}	6.64^{A}	5.46^{B}	4.70^{C}	0.19	< 0.001
IL-1β	2.04	2.10	2.38	1.98	2.14	0.13	0.290
IL-2	19.08	21.13	21.66	21.84	22.04	1.18	0.416
IL-4	1.48	1.32	1.26	1.30	1.17	0.08	0.159
IL-10	2.84^{A}	2.50^{B}	2.25^{BC}	2.08^{C}	2.09^{C}	0.09	< 0.001
Ileal mucosa							
TNF-α	4.44^{D}	4.75^{CD}	6.22^{A}	5.84^{AB}	5.32^{BC}	0.27	0.002
IL-1β	1.85	2.00	2.21	2.10	2.09	0.12	0.230
IL-2	19.98^{c}	20.50^{bc}	23.39^{a}	22.49^{ab}	20.23^{bc}	0.74	0.016
IL-4	1.38^{A}	1.04^{B}	0.80^{B}	0.85^{B}	0.97^{B}	0.09	0.003
IL-10	2.78^{A}	2.31^{B}	1.85^{B}	2.08^{B}	2.27^{B}	0.14	0.005

Note: TNF-α, tumor necrosis factor-α; IL-1β, interleukin-1β; IL-2, interleukin-2; IL-4, interleukin-4; IL-10, interleukin-4. SEM, standard error of the mean. $n = 4$. $^{A–D}$, different letters mean a statistical difference ($p < 0.01$). $^{a–c}$, different letters mean a statistical difference ($p < 0.05$).

Table S6. Ingredients composition and nutrient levels of the basal diet (%, as-fed basis).

Items	d 0–14	d 15–42
	Ingredients	
Corn	56.84	60.04
Soybean meal (46% CP)	11.80	17.60
Extruded full-fat soybean	10.00	8.00
Fermented soybean meal	8.00	5.00
Whey powder	4.00	-
Fish meal	2.00	-
Glucose	2.00	3.00
Wheat bran	1.50	2.00
Dicalcium phosphate	1.30	1.40
Limestone	0.70	0.80
Soybean oil	0.60	0.80

L-Lysine·HCl (78.8%)	0.35	0.38
Sodium chloride	0.26	0.32
DL-Methionine (98.5%)	0.15	0.16
Premix	0.50	0.50
Total	100.00	100.00
	Nutrient levels	
Digestible energy (MJ/kg)	14.29	14.21
Crude protein	21.09	20.23
Calcium	0.86	0.69
Total phosphorus	0.79	0.63
Lysine	1.36	1.33
Methionine+Cysteine	0.74	0.71

Note: The premix provided per kilogram diet: Vitamin A, 12,000 IU; Vitamin D3, 2,000 IU; Vitamin E, 30 IU; Vitamin K_3, 2.5 mg; Vitamin B_1, 2.5 mg; Vitamin B_2, 4 mg; Vitamin B_6, 3 mg; Vitamin B_{12}, 20 μg; Niacin, 40 mg; Pantothenic acid, 12.5 mg; Folic acid, 0.7 mg; Biotin, 0.07 mg; Choline chloride, 500 mg; Iron (from $FeSO_4$), 100 mg; Copper (from $CuSO_4$), 90 mg; Manganese (from $MnSO_4$), 80 mg; Zinc (from $ZnSO_4$), 80 mg; Iodine (from KI), 0.25mg; Selenium (from Na_2SeO_3), 0.3 mg. Digestible energy was a calculated value and others were measured values.

Table S7. The determination of mycotoxins in treatment diets (μg/kg).

Treatments	Addition	Measured values of mycotoxins					
		DON	ZEA	AFB_1	OTA	T-2	FB_1
			**d 0–14**				
CON	0	18.23	3.20	1.03			
DON0.25	250	282.62	4.74	0.58			
DON0.5	500	534.92	2.13	0.78		< LOQ	
DON1.0	1000	1097.51	5.31	0.95			
DON2.0	2000	1689.23	0.65	1.05			
			d 15–42				
CON	0	29.30	11.64	0.68			
DON0.25	250	312.70	7.81	0.77			
DON0.5	500	462.15	14.56	1.07		< LOQ	
DON1.0	1000	1043.46	10.75	0.75			
DON2.0	2000	1909.58	10.17	0.86			

Note: < LOQ, lower than the limit of quantification. The LOQ for OTA, T-2, and FB_1 were 0.5, 0.5, and 5 μg/kg, respectively.

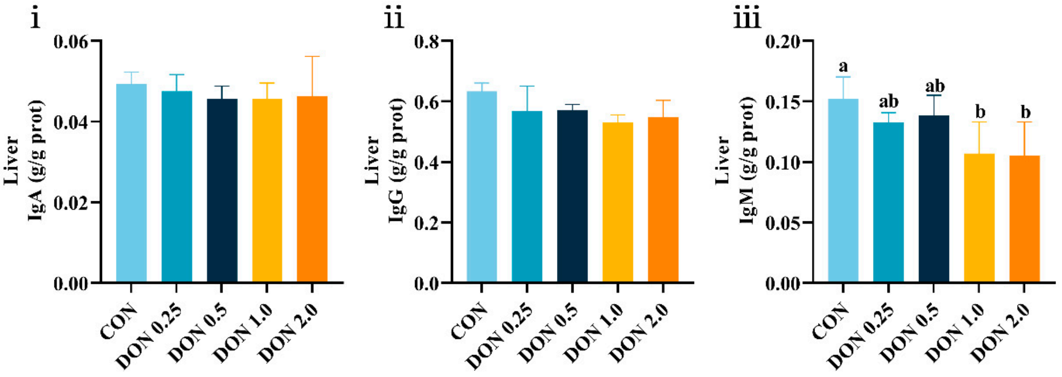

Figure S1. Effects of dietary DON exposure on liver immunoglobulin levels of piglets. (i) immunoglobulin A (IgA), (ii) Immunoglobulin G (IgG), (iii) Immunoglobulin M (IgM). Data are presented as means ± SD, n = 4. a–b, different letters mean a statistical difference ($p < 0.05$).

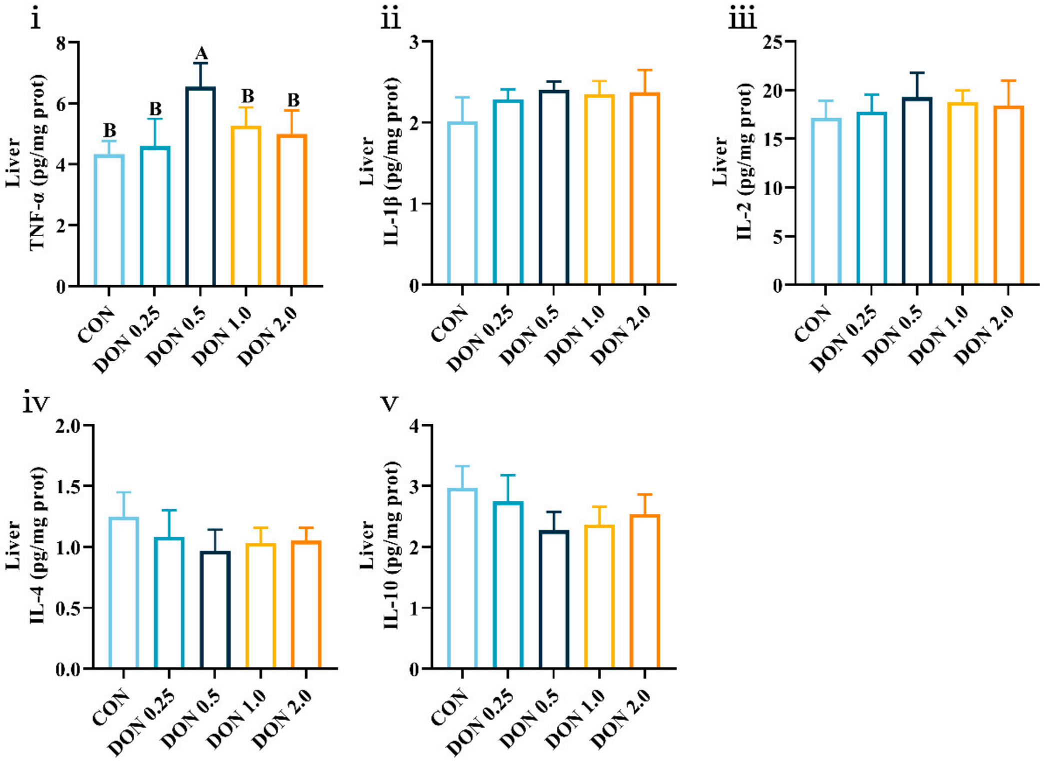

Figure S2. Effects of dietary DON exposure on liver immune cytokine levels of piglets. (i) Tumor necrosis factor-α (TNF-α), (ii) Interleukin-1β (IL-1β), (iii) Interleukin-2 (IL-2), (iv) Interleukin-4 (IL-4), and (v) Interleukin-10 (IL-10). Data are presented as means ± SD, n = 4. A–B, different letters mean a statistical difference ($p < 0.01$).
